# Photoaccumulation of Long‐Lived Reactive Electrons in a Microporous Ti(IV) Oxocluster‐Based Metal–Organic Framework for Light and Dark Photocatalysis

**DOI:** 10.1002/adma.202517595

**Published:** 2025-12-03

**Authors:** Shilin Yao, Katrin Heinzerling, Sam A. J. Hillman, Filip Podjaski, Tianhao He, Alberto García‐Baldoví, Yasmine Baghdadi, Khaled Dassouki, Hermenegildo García, Salvador Eslava, Nathalie Steunou, Soranyel Gonzalez‐Carrero, Sergio Navalón, Georges Mouchaham, Christian Serre, James R. Durrant

**Affiliations:** ^1^ Department of Chemistry Molecular Science Research Hub Imperial College London White City London W12 0BZ UK; ^2^ Institut des Matériaux Poreux de Paris ESPCI Paris Ecole Normale Supérieure CNRS PSL University Paris 75005 France; ^3^ Instituto de Tecnología Química (CSIC‐UPV) Agencia Estatal Consejo Superior de Investigaciones Científicas Universitat Politècnica de València Av. de los Naranjos s/n Valencia 46022 Spain; ^4^ Department of Chemical Engineering and Center for Processable Electronics Imperial College London London SW7 2AZ UK; ^5^ Institut Lavoisier de Versailles Université de Versailles St Quentin en Yvelines Université Paris Saclay 45 Avenue des Etats Unis Versailles 78035 France; ^6^ Institute of Molecular Science University of Valencia Paterna Valencia 46980 Spain; ^7^ Departamento de Química Universitat Politècnica de València Camino de Vera s/n Valencia 46022 Spain

**Keywords:** dark photocatalysis, long‐lived electrons, titanium metal–organic frameworks, transient absorption spectroscopy, water splitting

## Abstract

The microporous Ti_12_ oxocluster‐based metal–organic framework (MOF) MIP‐177(Ti)‐LT exhibits excellent stability and photoactivity, making it highly promising for photocatalysis. Using transient and photoinduced absorption spectroscopies, the behavior of reactive electrons in MIP‐177(Ti)‐LT across femtosecond‐to‐second timescales is investigated. The framework shows efficient charge separation and slow decay kinetics, with photogenerated charges persisting into the microsecond‐second (µs‐s) range and displaying higher yields and slower recombination than benchmark MOFs MIL‐125(Ti)‐NH_2_ and UiO‐66(Zr)‐NH_2_. Photogenerated holes oxidize water with an O_2_ yield of 335 µmol g^−1^ h^−1^ in the presence of electron scavengers. Under continuous irradiation, long‐lived electrons accumulate, further enhanced by a hole scavenger (methanol). Remarkably, these electrons persist over 48 h post‐excitation under argon, accompanied by a reversible white‐to‐black color change. The stored electrons remain redox‐active, efficiently reducing added O_2_ and methyl viologen. Dark addition of a Pt co‐catalyst to photocharged MIP‐177(Ti)‐LT induces H_2_ evolution at ≈300 µmol g^−1^ (≈58 C g^−1^), corresponding to an accumulated electron density of one electron per 12 Ti atoms. These results highlight the photocharging properties of MIP‐177(Ti)‐LT and its potential for sustainable photocatalytic applications.

## Graphical abstract

The microporous Ti_12_ oxocluster‐based MOF MIP‐177(Ti)‐LT exhibits effective charge separation and long‐lived photogenerated electrons persisting for hours, as revealed by spectroscopy. These electrons cause reversible color changes and enable dark H_2_ evolution and water splitting, demonstrating remarkable electron storage and redox capability, positioning MIP‐177(Ti)‐LT as a promising material for photocatalysis and solar energy storage.

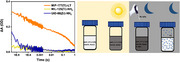

## 1 Introduction

Photocatalysis offers a promising solution to harnessing and storing solar energy, with applications including environmental remediation and artificial photosynthesis.^[^ ^1^ ^]^ Inorganic materials such as metal oxides are among the most established photocatalysts, being known for their excellent quantum efficiencies and stabilities, and are already finding commercial applications.^[^ ^2^ ^]^ However, these inorganic photocatalysts often face limitations such as restricted structural and spectral tunability; for example, high quantum efficiencies are typically only achieved with wide bandgap, UV‐absorbing metal oxide photocatalysts.^[^ ^3^ ^]^ In contrast, organic photocatalysts provide greater flexibility in tuning properties through chemical modifications, which improves control over their reactivity, absorption spectra, and selectivity.^[^ ^4^, ^5^ ^]^ However, organic photocatalysts generally exhibit lower structural robustness, stability, and efficiency.^[^ ^6^ ^]^ In particular, they often suffer from short‐lived photoexcited states (excitons), which lead to rapid recombination and can significantly limit their overall photocatalytic performance.^[^ ^7^ ^]^

Metal–organic Frameworks (MOFs) are a class of ordered porous materials that bridge the gap between inorganic and organic materials. They are generally composed of metal‐oxo clusters (or chains, layers) connected by organic ligands, and incorporate features of both material classes. MOFs offer a high degree of tunability,^[^ ^8^ ^]^ as both the inorganic nodes and ligands can be selected and tailored to achieve desired pore sizes, framework structures, and photophysical properties for targeted photocatalytic reactions.^[^ ^9^ ^]^ Additionally, the porous nature of MOFs provides a high specific surface area and numerous exposed redox‐active sites, facilitating reactant accessibility to these active sites.^[^ ^10^ ^]^ For example, Ti‐based MOFs have demonstrated excellent photoactivity and selectivity in photocatalytic organic transformations, as well as in more kinetically challenging reactions such as overall water splitting, achieving through appropriately functionalized structural design.^[^ ^11^, ^12^, ^13^, ^14^ ^]^ However, despite these apparent advantages, MOFs reported to date typically exhibit lower photocatalytic efficiencies than optimized inorganic or organic photocatalysts, limiting their practical applications.^[^ ^15^, ^16^, ^17^, ^18^ ^]^

Charge generation, separation, and recombination kinetics are critical determinants of photocatalytic efficiencies.^[^ ^19^ ^]^ In many photocatalyst systems, such as metal oxides,^[^ ^19^ ^]^ carbon nitrides,^[^ ^20^ ^]^ and conjugated polymers,^[^ ^7^, ^21^ ^]^ understanding and optimising these kinetic processes is closely correlated with advancements in photocatalytic performance. However, despite their importance, the photoexcited state kinetics of MOFs photocatalysts have received relatively limited attention to date.^[^ ^22^, ^23^, ^24^ ^]^ As such, photocatalytic structure‐activity relationships of MOFs are currently only poorly understood, particularly in terms of tuning photoexcited state dynamics.^[^ ^25^ ^]^ This knowledge gap hinders the development of more efficient MOF‐based photocatalyst systems and highlights the need for focused research on the photophysics of these materials to unlock their full potential in photocatalytic applications.

Photophysical studies of MOFs, particularly Ti‐MOFs and their photoresponsive derivatives,^[^ ^26^ ^]^ have focused primarily on charge generation and separation.^[^ ^27^ ^]^ Time‐resolved optical investigations indicate that in some MOFs, photogenerated electrons can localize on inorganic clusters, while holes often reside on the organic linkers; modifying these organic linkers has been shown to enhance charge separation. For example, in the microporous Ti terephthalate MIL‐125(Ti), the introduction of amino functional groups (─NH_2_) in the organic linker significantly improves charge separation^[^ ^28^ ^]^ and reduces the optical bandgap from 3.6 to 2.6 eV.^[^ ^29^ ^]^ However, even with these modifications, many MOFs, including MIL‐125(Ti)‐NH_2_ (usually called MIL‐125‐NH_2_(Ti) in literature), exhibit limited apparent quantum efficiencies for photocatalysis (for example, 5% at 420 nm for hydrogen evolution in the presence of a sacrificial electron donor,^[^ ^30^ ^]^ and relatively poor stability.^[^ ^31^ ^]^ This efficiency limitation reflects a broader trend observed across various MOF systems, where spectroscopic studies have found that most neat MOFs are rather inefficient at generating charges that live long enough to drive photocatalytic reactions.^[^ ^7^, ^32^, ^33^, ^34^, ^35^ ^]^ This limitation can be partially mitigated by using sacrificial electron donors/acceptors and co‐catalysts for MOF‐driven photocatalysis.^[^ ^36^ ^]^ For example, Cu‐modified MIL‐125(Ti)‐NH_2_ has been demonstrated to show an ≈30‐fold increase in excited‐state lifetime compared to neat MIL‐125(Ti)‐NH_2_, correlating with a similar enhancement in H_2_ evolution activity.^[^ ^37^ ^]^ Comparison with other photocatalyst systems^[^ ^7^, ^19^, ^20^, ^21^ ^]^ clearly indicates that identifying MOFs with the intrinsic ability to generate long‐lived charges following photoexcitation offers the potential to significantly enhance their photocatalytic efficiencies.

**Figure 1:**
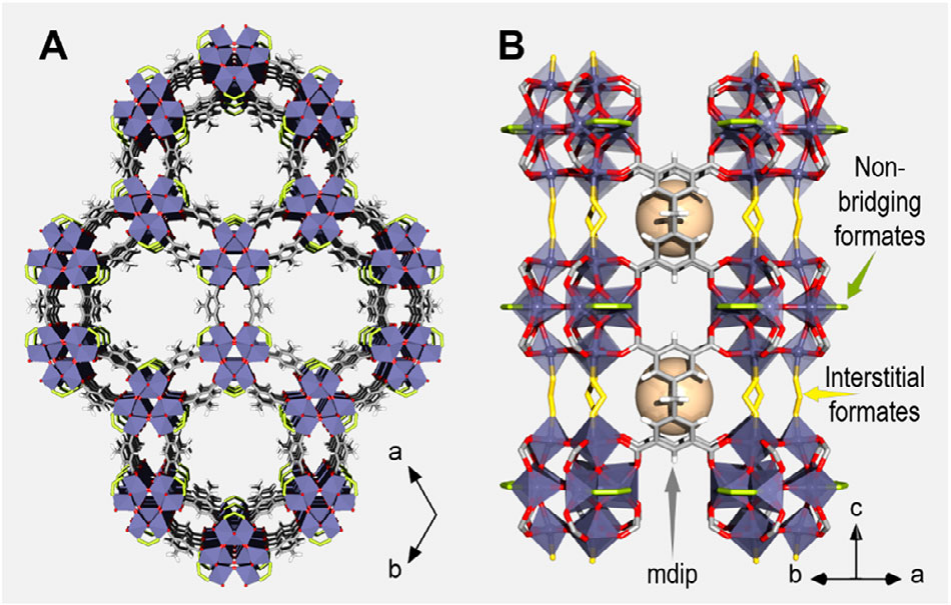
Crystal structure of MIP‐177(Ti)‐LT showing a) the 3‐D framework along the *c*‐axis, and b) a close‐up on the Ti_12_O_15_‐clusters linked via mdip and interstitial formates. Color codes: TiO_6_, purple polyhedra; C, grey; O, red; H, white; interstitial formates, yellow; non‐bridging formates, green. Spheres shown in tan represent the small pocket between mdip linkers.

Recently, the microporous Ti tetracarboxylate MIP‐177(Ti)‐LT^[^ ^38^ ^]^ (**Figure** **1**) has shown promising photocatalytic activity, particularly for hydrogen production from formic acid oxidation without additional sacrificial agents or co‐catalysts (MIP: Institute of Porous Materials, Paris; LT: structure obtained at relatively Low Temperature).^[^ ^39^, ^40^ ^]^ This MOF achieved an apparent quantum yield of 22% in the UV, which is 3–4 times higher than that observed in other more widely studied MOFs such as MIL‐125(Ti) and UiO‐66(Zr). MIP‐177(Ti)‐LT consists of Ti_12_O_15_ clusters connected by mdip (where mdip is methylene di‐isophthalate, with its carboxylate groups ligating the metal oxide clusters) and with interstitial formates along the alignment axis of the clusters (the *c*‐axis) (Figure 1b),^[^ ^38^ ^]^ forming a porous 3D framework endowed with 1D channels along the *c*‐axis of ≈1 nm diameter. The mdip linker was selected to balance rigidity and flexibility through its methylene spacer, enabling efficient connection of the large Ti_12_O_15_ clusters into a stable 3D network with aligned 1D channels and short inter‐cluster distances that can facilitate charge transport.^[^ ^38^ ^]^ Remarkably, the MIP‐177(Ti)‐LT exhibits a unique Ti_12_O_15_ oxocluster with the highest O/Ti ratio reported for Ti‐cluster‐MOFs, together with the shortest inter‐cluster distance (i.e., one formate), promoting close proximity between adjacent clusters along a single direction.^[^ ^38^ ^]^ Moreover, this synthetically‐scalable MOF exhibits excellent chemical stability, maintaining its structure even under highly corrosive (acidic) conditions such as exposure to aqua regia and concentrated H_3_PO_4_.^[^ ^39^, ^41^ ^]^ In contrast to the distinct tubular architecture of MIP‐177(Ti)‐LT, the extensively investigated UiO‐66(Zr)‐NH_2_ (usually called UiO‐66‐NH_2_ (Zr) in literature) (Figure S1a, Supporting Information) and MIL‐125(Ti)‐NH_2_ (Figure S1b, Supporting Information) are characterized by 3D frameworks (with **fcu** topology) composed of interconnected tetrahedral and octahedral cages, featuring pore sizes of 7.5/12 and 6/12 Å, respectively. In both materials, the frameworks are built by connecting metal oxo/hydroxo clusters ‐specifically Zr_6_O_4_(OH)_4_ in UiO‐66(Zr)‐NH_2_ and Ti_8_O_8_(OH)_4_ in MIL‐125(Ti)‐NH_2_ with aminoterephthalate organic linkers. In contrast to MIP‐177(Ti)‐LT, their constitutive metal (IV) oxoclusters are relatively small and well‐separated from each other by the organic ligand (Figure S1, Supporting Information).

Despite the promising photocatalytic potential shown for MIP‐177(Ti)‐LT, understanding of its photophysical properties remains limited.^[^ ^23^ ^]^ In this study, we employ transient absorption spectroscopy (TAS) and photoinduced absorption spectroscopy (PIAS) to investigate the charge carrier dynamics within MIP‐177(Ti)‐LT across timescales ranging from femtoseconds to seconds. These kinetics are contrasted with those of MIL‐125(Ti)‐NH_2_ and UiO‐66(Zr)‐NH_2_ to gain insight into the structure‐activity relationship for these MOF photocatalysts. We find that MIP‐177(Ti)‐LT exhibits an excellent ability to separate photogenerated charges, leading in particular to the accumulation of long‐lived electrons under an inert atmosphere. Notably, these long‐lived (up to days) electrons are shown to be reactive, capable of driving the hydrogen evolution reaction in the dark following photoinduced electron accumulation. This electron accumulation is correlated with water oxidation to yield molecular oxygen, resulting in water splitting with temporally separated oxygen and hydrogen evolution. Enhanced electron accumulation is observed in the presence of a hole scavenger methanol, resulting in an electron accumulation capacity of ≈1 reactive electron per Ti_12_O_15_ cluster under prolonged illumination. These insights highlight MIP‐177(Ti)‐LT as a promising material for efficient and sustainable photocatalytic processes and, moreover, indicate its further potential as a photochargable material.

## 2 Results

### 2.1 Steady‐State Absorption and Emission of MIP‐177(Ti)‐LT

**Figure 2:**
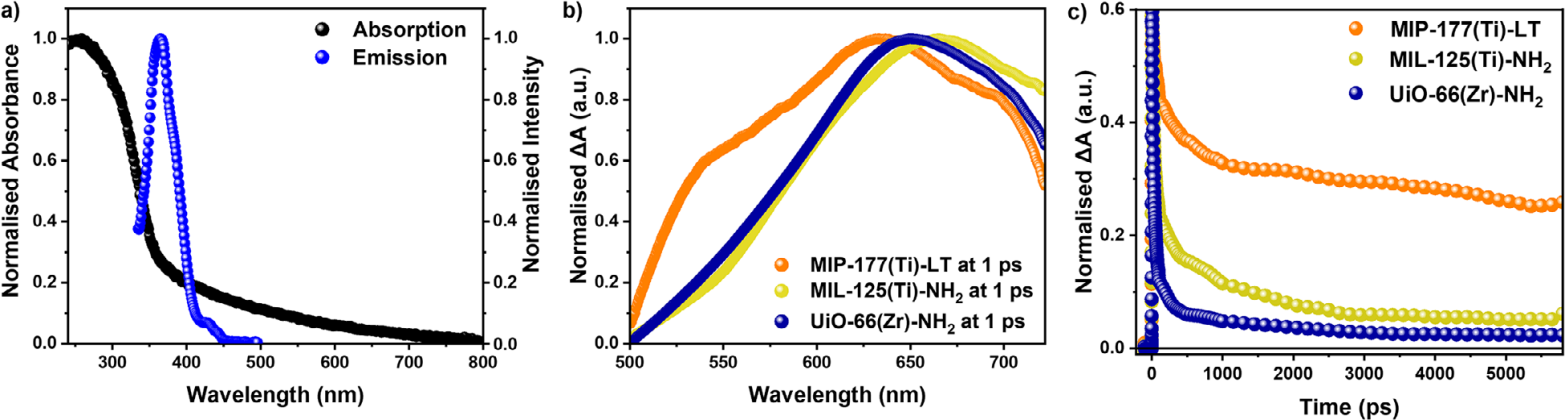
a) Normalized steady‐state UV–vis absorption spectrum (black) of 0.1 mg mL^−1^ MIP‐177(Ti)‐LT water suspension measured in 2 mm cuvette, and normalized emission spectrum (blue, λ_ex_ = 320 nm) of 0.1 mg mL^−1^ MIP‐177(Ti)‐LT water suspension measured in 1 cm PL cuvette. b,c) Comparison of normalized fs‐TAS spectra and normalized fs‐TAS kinetics (probed at 650 nm, normalized at 0.78 ps of MIP‐177(Ti)‐LT, MIL‐125(Ti)‐NH_2_, and UiO‐66(Zr)‐NH_2_ measured on film in water. Excitation wavelength of 320 nm (pulsed laser intensity: 640 uJ cm^−2^, frequency: 500 Hz).

The steady‐state UV–vis absorption spectrum of MIP‐177(Ti)‐LT water suspension exhibits an absorption up to ≈355 nm (**Figure** **2**a; Figure S2a, Supporting Information), in agreement with previous literature.^[^ ^38^, ^39^, ^42^, ^43^, ^44^ ^]^ A small tail in the spectrum is ascribed to the intrinsic scattering characteristics of the material. The photoluminescence (PL) spectrum, upon 320 nm excitation, shows an emission peak within the range of 330–430 nm, with a maximum at 365 nm (Figure 2a; Figure S2b, Supporting Information). These absorption and emission features of MIP‐177(Ti)‐LT are comparable with those of other reported Ti‐based MOFs with wide bandgap linkers and are analogous to those reported for bulk titania.^[^ ^45^, ^46^, ^47^ ^]^

### 2.2 TAS Study of Charge Carrier Dynamics in MIP‐177(Ti)‐LT

To understand the photophysical properties of MIP‐177(Ti)‐LT, an investigation into its charge carrier dynamics was conducted using TAS across a range of timescales from femtoseconds to seconds. Ultrafast Transient Absorption Spectra (fs‐TAS) of MIP‐177(Ti)‐LT film in water under Ar atmosphere were obtained with an excitation wavelength of 320 nm, revealing a broad positive photoinduced absorption in the range of 500–725 nm, centerd ≈630 nm (Figure 2b; Figure S3a, Supporting Information). Similar photoinduced absorption was observed for MIL‐125(Ti)‐NH_2_ and UiO‐66(Zr)‐NH_2_ films under similar conditions (Figure 2b; Figure S3a–c, Supporting Information), in agreement with previous studies of these two benchmark MOFs.^[^ ^28^, ^48^ ^]^ However, it is notable that in terms of the decay dynamics of this photoinduced absorption, MIP‐177(Ti)‐LT shows much slower decay dynamics (half‐life ≈50 ps) than these reference MOFs (Figure 2c; Figure S3a–c, Supporting Information). MIL‐125(Ti)‐NH_2_ and UiO‐66(Zr)‐NH_2_ both exhibit rapid decay of their photoinduced absorption (half‐life ≈10 ps), with respectively circa 96% and 98% decay within 5 ns (Figure 2c; Figure S3a–c, Supporting Information), consistent with previously reported fast decay dynamics for these and related MOFs.^[^ ^48^, ^49^ ^]^ In contrast, the photoinduced absorption signal for MIP‐177(Ti)‐LT exhibited much slower decay dynamics, with circa 25% of the initial signal remaining at 5 ns. These decay kinetics were observed to be laser intensity‐independent (Figure S3d,e, Supporting Information). The slower decay kinetics in MIP‐177(Ti)‐LT clearly suggest a more effective charge separation, resulting in suppressed charge recombination and a higher yield of long‐lived charges.

**Figure 3:**
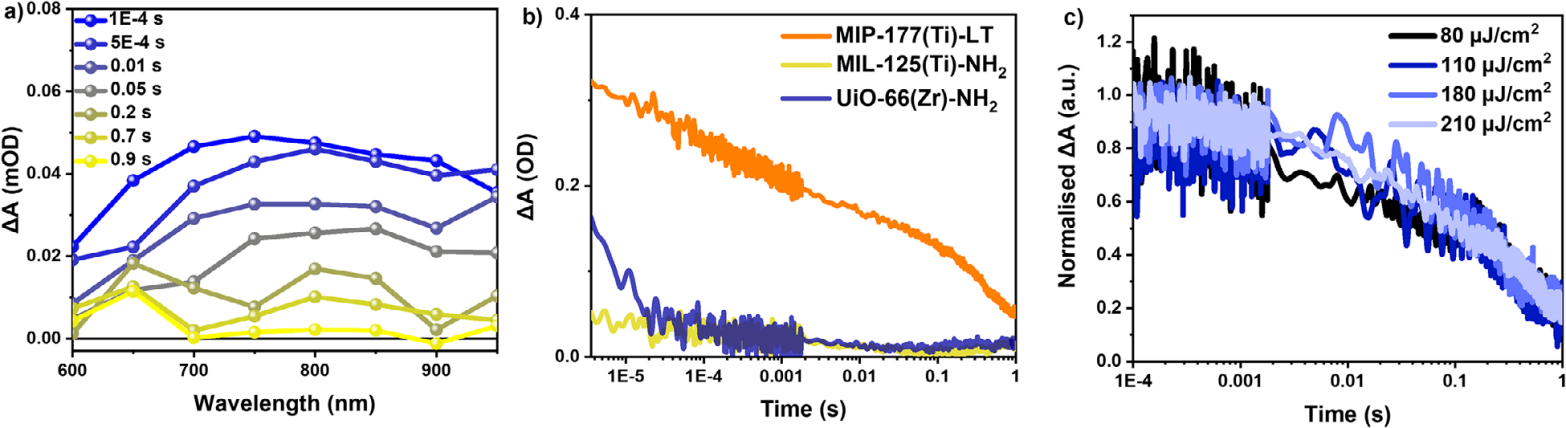
a) DR‐TAS spectra of MIP‐177(Ti)‐LT measured on film in water, excited by 355 nm pulsed laser (intensity: 280 µJ cm^−2^, frequency: 1 Hz). b) Comparison of DR‐TAS kinetics MIP‐177(Ti)‐LT, MIL‐125(Ti)‐NH_2_, and UiO‐66(Zr)‐NH_2_ water suspension (6.4 mg mL^−1^), probed at 650 nm (excitation wavelength: 355 nm, intensity: 270 µJ cm^−2^, frequency: 1 Hz). c) Normalized DR‐TAS kinetics of MIP‐177(Ti)‐LT water suspension, probed at 650 nm, excited by 355 nm pulsed laser, with intensity range from 80 to 210 µJ cm^−2^.

We next tracked the µs‐s decay kinetics on MOF films using diffuse‐reflectance TAS (DR‐TAS), with an excitation wavelength of 355 nm and an intensity of 280 µJ cm^−2^ (no change in kinetics between excitation wavelengths, Figure S4a, Supporting Information). The MIP‐177(Ti)‐LT film in water under Ar atmosphere exhibits a broad spectrum (600–950 nm) (**Figure** **3**a), similar to that observed in fs‐TAS above, which is assigned to long‐lived photogenerated charges. It is notable that the decay dynamics of these long‐lived charges in MIP‐177(Ti)‐LT are surprisingly slow, with a decay half‐life for these transients of ≈20 ms following pulsed excitation (Figure 3b; Figure S4b, Supporting Information). It is also apparent from this figure that both MIL‐125(Ti)‐NH_2_ and UiO‐66(Zr)‐NH_2_ exhibit almost negligible signals on this µs‐s under matched excitation conditions, with only a small, short‐lived (<10 µs) signal being observed for UiO‐66(Zr)‐NH_2_. The 20 ms half‐life observed herein for MIP‐177(Ti)‐LT is also noteworthy when compared to lifetimes of long‐lived charges reported previously in other MOFs – ranging from 250 ps,^[^ ^50^ ^]^ 25 ns,^[^ ^51^ ^]^ 2 µs,^[^ ^52^ ^]^ to 8 µs,^[^ ^34^ ^]^ and to the maximum reported MOF charge half‐life of 200 µs.^[^ ^53^ ^]^ Such prolonged photogenerated charge lifetimes have been reported in metal oxides, but generally only under strong anodic bias or in the presence of a sacrificial scavenger.^[^ ^54^ ^]^ To the best of our knowledge, the ability of MIP‐177(Ti)‐LT to generate such long‐lived charges in water without added scavenger or applied bias is quite unique compared to other MOF (or metal oxide) studied to date and may therefore hold promise for its application as a photocatalyst.

The decay kinetics of the long‐lived photogenerated charges in MIP‐177(Ti)‐LT illustrated in Figure 3b, extend over several orders of magnitude. Such “dispersive” kinetics do not correspond to ideal first or second‐order kinetics. Similar dispersive decay behavior has been reported in a range of photocatalyst materials, including metal oxides, carbon nitrides, and organic nanoparticles, and have been typically assigned to the impact of charge trapping in these materials.^[^ ^55^ ^]^ Charges trapped in relatively shallow traps can undergo reversible trapping/de‐trapping and thus faster decay kinetics, whilst more deeply trapped carriers exhibit slower recombination kinetics.^[^ ^56^ ^]^ It appears likely that the dispersive kinetics observed herein for MIP‐177(Ti)‐LT also result from the recombination kinetics of trapped charges. Supporting this conclusion, the decay kinetics in MIP‐177(Ti)‐LT were observed to be independent of excitation intensity (Figure 3c; Figure S4d, Supporting Information), as has been observed in other photocatalysts in the presence of charge trapping.^[^ ^57^ ^]^

### 2.3 Spectroelectrochemical (SEC) Analysis of Electron Energetics in MIP‐177(Ti)‐LT

To investigate the energetics of charge accumulation in MIP‐177(Ti)‐LT, SEC was employed on a MIP‐177(Ti)‐LT film deposited on a transparent FTO electrode, using an Ag/AgCl reference electrode and a 0.1 mol L^−1^ Na_2_SO_4_ aqueous solution under N_2_ atmosphere (see details in Section S1.1.4, Supporting Information, and the experimental flow and setup illustrated in Figure S5a,b, Supporting Information).^[^ ^58^ ^]^ This method allows measurement of the absorption of electrons injected into MIP‐177(Ti)‐LT by an applied electrical bias in the dark rather than the photoexcitation conditions employed above. When a negative potential was applied to the MIP‐177(Ti)‐LT film, a broad positive absorbance signal was observed, indicating electron accumulation within the material (Figure S6a, Supporting Information). No corresponding signal was detected under positive potentials ranging from 0 to +1.7 V (Figure S6b, Supporting Information), suggesting either that holes present in MIP‐177(Ti)‐LT do not exhibit absorption within the 450–820 nm range explored, or are insufficiently mobile to accumulate across the electrode. Notably, the SEC absorbance spectrum under negative bias closely resembles both the fs‐TAS and the DR‐TAS spectrum (compare Figure S3a, Supporting Information; Figures 2b and 3a), indicating that these spectra can be assigned to electrons on MIP‐177(Ti)‐LT. This result is consistent with the electron absorption signal being associated with the reduction of Ti⁴⁺ to Ti^3^⁺, as observed in bulk and nanocrystalline TiO_2_, where Ti^3+^ states are observed to absorb in the visible and near‐IR ranges.^[^ ^59^, ^60^ ^]^

**Figure 4:**
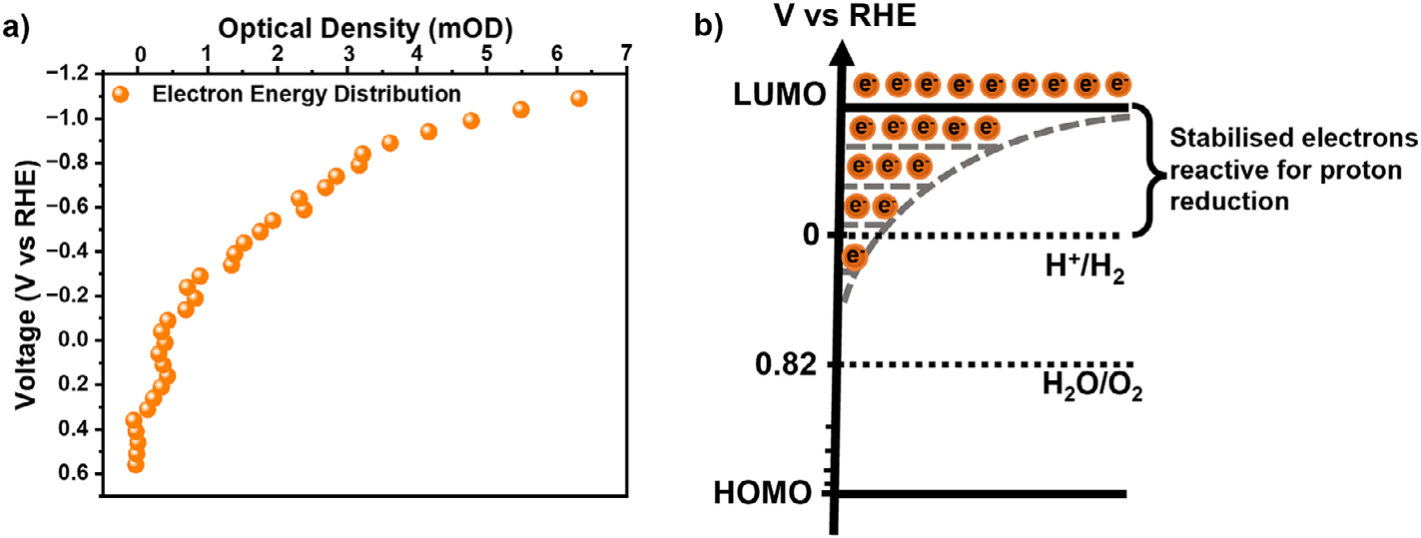
a) Spectroelectrochemical analysis of MIP‐177(Ti)‐LT film absorption at 650 nm as a function of applied potential. Recorded on film deposited on FTO glass, in 0.1 mol L^−1^ Na_2_SO_4_ aqueous solution with applied potential, from +0.61 to −1.09 V versus RHE. b) Schematic representation of electron trap energy distribution estimated on MIP‐177(Ti)‐LT from these SEC data.

The SEC signal assigned to MIP‐177(Ti)‐LT electrons increases over a broad range of applied bias V from circa +0.2 to −1.1 V versus RHE (**Figure** **4**a; Figure S6a, Supporting Information). This increase can be fitted reasonably to an exponential ([e^−^]∼µ exp (‐ V/E_ch_)) with a characteristic energy E_ch_≈500 meV (Figure S6c, Supporting Information). Such non‐ideal behavior (i.e.,: E_ch_>k_B_T) has also been reported previously in titania and assigned to an exponential tail of trap states extending below its LUMO edge.^[^ ^61^ ^]^ As such, it appears likely that the non‐ideal behavior we observe herein for MIP‐177(Ti)‐LT is also indicative of significant electron trapping in this material, as indicated in Figure 4b, and in agreement with our observation of dispersive charge recombination kinetics, as discussed above. It should be noted that the energetic magnitude of this trapping (as quantified by E_ch_ = 500 meV) is greater than is typically reported for titania (E_ch_≈70 meV),^[^ ^62^ ^]^ which may be indicative of greater chemical inhomogeneity in MIP‐177(Ti)‐LT. Despite this deeper trapping, it is also apparent that only the deepest traps lie at potentials more positive than 0 V_RHE_, with nearly all trap levels lying at significantly more negative potential. This suggests that Ti^3+^ states in MIP‐177(Ti)‐LT are significantly more reducing than those typically observed in titania,^[^ ^39^, ^63^ ^]^ most likely due to the presence of carboxylate ligands from the organic linkers. Furthermore, the high surface area and abundance of accessible active sites in MIP‐177(Ti)‐LT may reduce the need for long‐range charge mobility under reaction conditions, compared with dense oxides such as TiO_2_, and may therefore help maintain high electron reactivity despite the presence of deeper traps.

### 2.4 PIAS Investigtation of Long‐Lived Electrons and Black MIP‐177(Ti)‐LT

**Figure 5:**
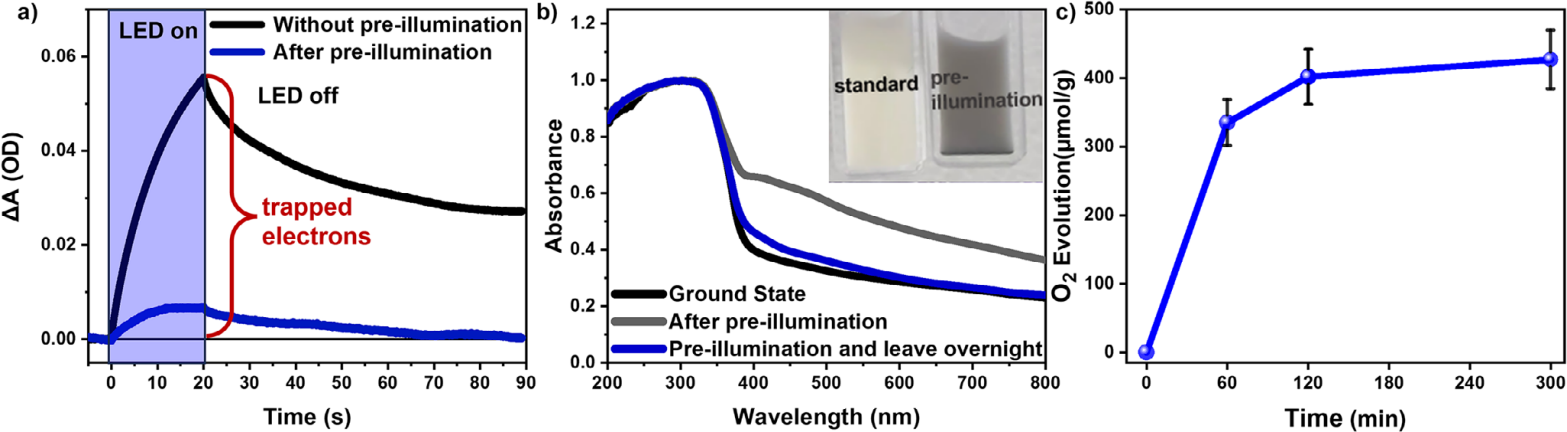
a) Comparison of the PIAS kinetics probed at 650 nm of MIP‐177(Ti)‐LT suspension (3.2 mg mL^−1^) before (black line) and after (blue line) of continuous LED pre‐illumination (1 h, 365 nm, 13.64 mW cm^−2^). b) Comparison of the UV–vis absorption spectra of MIP‐177(Ti)‐LT before (grey) and after (black) pre‐illumination (1 h), and then after leaving the sample overnight, showing a return to the ground state (blue). Insert: photos depicting the color change assigned to electron accumulation of the MIP‐177(Ti)‐LT suspension before (left) and after (right). c) Photocatalytic oxygen evolution from water oxidation in the presence of 1.5 mol L^−1^ (NH_4_)_2_[Ce(NO_3_)_6_] as electron scavenger under 1 sun illumination.

PIAS was employed to investigate the MIP‐177(Ti)‐LT electron dynamics under operando illumination conditions and on a catalytic timescale. A continuous pulsed LED was used for light excitation (i.e., quasi‐steady state) rather than a short (fs or ns) pulsed laser excitation. The PIAS spectrum of MIP‐177(Ti)‐LT dispersed in water under Ar atmosphere was obtained by illumination of the sample during 20 s (365 nm LED, intensity of 13.6 mW cm^−2^), followed by a 70 s period decay recorded without light illumination (LED‐off decay, **Figure** **5**a, black; Figure S7a, Supporting Information). The PIAS spectrum closely resembles the absorption features observed in TAS spectra described above, exhibiting a broad positive absorption from 500 to 1000 nm, consistent with the accumulation of long‐lived photogenerated electrons (Ti^3+^ species) in MIP‐177(Ti)‐LT. In contrast, both benchmark Ti/Zr MOFs exhibited very small PIAS signals (Figure S7b, Supporting Information), in agreement with our TAS studies above. We can conclude that under this quasi‐continuous irradiation for 20 s, a significant fraction of photogenerated electrons persist in MIP‐177(Ti)‐LT for at least tens of seconds (Figure 5a, black).

Our observation of long‐lived electron accumulation in MIP‐177(Ti)‐LT can be attributed, at least in part, to the presence of a broad, energetic distribution of electron trap states, as evidenced by our SEC data above. Further evidence to support this conclusion comes from the effect observed under pre‐illumination conditions in our PIAS data (Figure 5a). Pre‐illumination is expected to fill deep trap states, reducing the potential for subsequent photoexcitation to generate additional deeply trapped, and therefore long‐lived, electrons, as has been reported previously in both metal oxide and carbon nitride materials exhibiting deep charge trapping.^[^ ^64^, ^65^ ^]^ Consistent with this expectation, pre‐illumination (1 h with continuous 365 nm LED, 13.6 mW cm^−^^2^) resulted in a circa tenfold reduction in the amplitude of signals in subsequent PIAS measurements (Figure 5a; Figure S7c, Supporting Information). This is indicative of the suppression of the yield of additional long‐lived electrons and supports our conclusion that these electrons are trapped in the trap states identified in our SEC data.

The pronounced long‐lasting electron accumulation observed in our PIAS data was further evidenced by a visible color change in the material, transitioning from white to black (Figure 5b, insert, top). The material returned to its original white state after being left overnight in the dark (Figure 5b, insert, bottom), likely due to oxygen leakage into the cuvette. Further studies using spiral cap cuvettes with improved sealing extended the duration of the dark coloration to over 48 h, as shown in Figure S9d (Supporting Information). UV–vis absorption spectra further supported this transformation, showing increased absorption from 400 to 800 nm with electron (Ti^3+^) accumulation (Figure 5b). This coloration resembles the absorption feature of “black” titanium oxide Ti_4_O_7_ reported in the literature,^[^ ^66^, ^67^ ^]^ which was also assigned to the presence of Ti^3+^ species.^[^ ^60^, ^68^ ^]^ Electron paramagnetic resonance (EPR) spectra further confirmed the formation of Ti^3+^ during illumination (Figure S8a, Supporting Information), characterized by the growth of a signal with a g value of 1.92, accompanied by another EPR signal appearing at a g value ≈2, attributable to holes trapped on oxygen (section S3.3, Supporting Information).^[^ ^69^ ^]^

Our observation of remarkably long‐lived electron accumulation in MIP‐177(Ti)‐LT is indicative of a pathway to extract photogenerated holes from the system. Notably, this photoinduced electron accumulation was reversible (Figure S9c, Supporting Information), suggesting it is not associated with any irreversible degradation of the MOF. In order to investigate possible pathways for hole extraction in the absence of hole scavengers, we undertook measurements of oxygen evolution in water with the addition of (NH_4_)_2_[Ce(NO_3_)_6_] (1.5 mol L^−1^) as an electron scavenger. Under ≈1 sun irradiation (150 W Xe lamp with AM 1.5 cut‐off filter, 100 mW cm^−2^, see details in the Section S1.4.1, Supporting Information), O_2_ evolution was detected, with a yield of 335 ± 33 µmol g^−1^ in 1 h (Figure 5c). Consistent with this result, photoinduced overall water splitting (OWS) – conducted without the use of scavengers and co‐catalysts – was also observed for MIP‐177(Ti)‐LT, with H_2_ and O_2_ production of 70 and 30 µmol g^−1^ in 1 h, respectively (Figure S8c, Supporting Information). An additional photocatalytic OWS experiment using label H_2_^18^O and analysis of the gas headspace via gas chromatography‐mass spectrometry revealed a peak at m/z 36, corresponding to ^18^O_2_ from water oxidation (Figure S8d, Supporting Information). To further investigate the behavior of photogenerated holes in MIP‐177(Ti)‐LT, we performed additional photocatalytic oxygen evolution experiments, monitoring O_2_ evolution in situ using a Clark electrode using Na_2_S_2_O_8_ (0.1 mol L^−1^) as an electron scavenger. As shown in Figure S8b (Supporting Information), O_2_ evolution through water oxidation was observed, with its temporal profile monitored in real‐time via the Clark electrode. These photocatalytic results indicate that in the absence of an added hole scavenger, the most likely pathway for hole extraction, enabling long‐lived electron accumulation, is water oxidation by MIP‐177(Ti)‐LT valence band holes.

**Figure 6:**
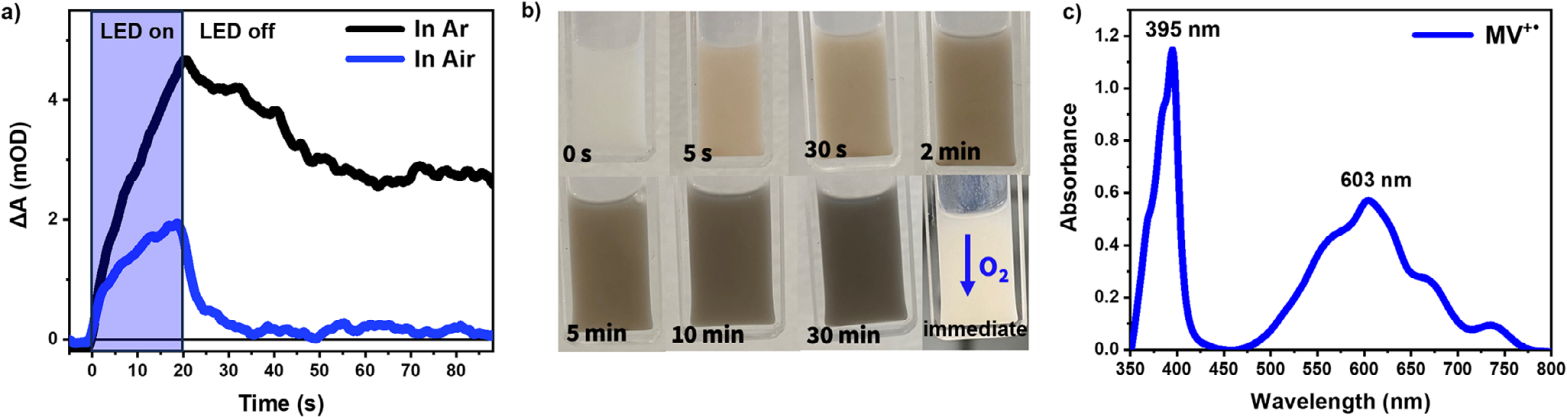
a) PIAS kinetics comparison of a MIP‐177(Ti)‐LT film in water in Ar (black) and in Air (blue), excited by 365 nm LED (13.64 mW cm^−2^), probed at 650 nm, measured in reflectance. b) Photos depicting the color change of MIP‐177(Ti)‐LT suspension results from photoaccumulation of electrons after 0s, 5 s, 30 s, 2 min, 5 min, 10 min and 30 min of pre‐illumination, in the presence of methanol as hole scavenger and in Ar. Subsequently, upon exposure to air, an immediate color change back to white was induced. c) UV–vis absorption of MV^+^ **^.^** after adding 50 µL 0.1 mmol mL^−1^ methyl viologen aqueous solution into the 6.4 mg mL^−1^ pre‐illuminated MIP‐177(Ti)‐LT suspension.

Improved electron accumulation was observed in the presence of methanol (10% by volume) as a hole scavenger to remove the photogenerated holes. The color change in MIP‐177(Ti)‐LT under LED illumination was observed more rapidly in the presence of methanol (**Figure** **6**b), indicative of more efficient charge accumulation relative to that observed in the absence of a scavenger (5 min vs 30 min, respectively) (Figure S9b, Supporting Information). This can be assigned to methanol addition significantly enhancing the extraction of photogenerated holes from the MIP‐177(Ti)‐LT, and thereby increasing the electron accumulation process, providing further confirmation of our assignment of the long‐lived coloration of MIP‐177(Ti)‐LT under irradiation to long‐lived electron accumulation.

### 2.5 Highly Reactive Long‐Lived Electrons in MIP‐177(Ti)‐LT

Our µs‐s DR‐TAS and SEC data reported above suggest that the long‐lived electrons we observe to accumulate in MIP‐177(Ti)‐LT under continuous irradiation (365 nm) are most likely trapped within the MOF. A key consideration is therefore whether these trapped electrons retain sufficient energy to drive interfacial reactions, as has been previously considered in other photocatalytic systems.^[^ ^20^, ^56^, ^70^, ^71^, ^72^ ^]^

Evidence for the reactivity of the long‐lived accumulated electrons in MIP‐177(Ti)‐LT comes from their quenching in the presence of molecular oxygen.^[^ ^73^ ^]^ The PIAS kinetics of MIP‐177(Ti)‐LT film in water are strongly quenched when exposed to air (Figure 6a), with a smaller electron population and a much faster decay (half‐life of ≈2 s) relative to the film in Ar atmosphere (half‐life longer than 70 s). This quenching of long‐lived electron accumulation is assigned to these electrons reducing molecular oxygen, as has been previously widely reported for TiO_2_ photocatalysts.^[^ ^74^ ^]^ This quenching of PIAS signal in the presence of oxygen was observed across the entire spectral range measured (Figure S7e, Supporting Information). This effect was also apparent by visual observation, with exposure of a pre‐illuminated MIP‐177(Ti)‐LT suspension resulting in rapid (<1 s) decoloration of the suspension from black to white (Figure 6b; Figure S9a, Supporting Information), highlighting the high reactivity of the accumulated electrons with oxygen. Subsequent LED illumination turns the MIP‐177(Ti)‐LT black again (Figure S9c, Supporting Information), demonstrating that the electron accumulation is highly reversible and, therefore, not caused by the degradation of the MIP‐177(Ti)‐LT.

The oxygen reduction reaction, generating O_2_^−^ or other reactive oxygen species, is energetically favourable, with a reduction potential under neutral conditions and at room temperature of +0.82 V versus RHE,^[^ ^75^ ^]^ significantly more positive than the LUMO edge of MIP‐177(Ti)‐LT, as previously reported in the literature,^[^ ^39^ ^]^ and more positive than almost all of the electron trap state energies determined from our SEC data above. This energy difference allows oxygen to effectively capture electrons from the LUMO of MIP‐177(Ti)‐LT. We turn now to a more energetically challenging reduction reaction, the reduction of the electron acceptor methyl viologen (MV^2+^, reduction potential ‐0.046 V vs RHE,^[^ ^76^ ^]^) studied in the presence of methanol so as to maximize the electron photoaccumulation. After 10 min of pre‐illumination (365 nm LED) in the presence of methanol, the addition of methyl viologen (MV^2+^) water solution induced an immediate color change in MIP‐177(Ti)‐LT from black to blue (Figure S10a, Supporting Information), accompanied by a distinct UV–vis absorption change corresponding to the absorption of MV^+.^ (Figure 6c; Figure S10b,c, Supporting Information). Employing the previously reported extinction coefficient of MV^+.^ (13 500 L mol^−1^ cm^−1^ at 600 nm),^[^ ^70^ ^]^ this we estimated that ≈33 µmol electrons were accumulated per gram MIP‐177(Ti)‐LT during 10 min of illumination (365 nm LED, intensity of 13.6 mW cm^−2^). This clearly demonstrates the efficiency of long‐lived electron accumulation in MIP‐177(Ti)‐LT in the presence of hole scavenger. It further demonstrates that the photoaccumulated long‐lived electrons retain sufficient reactivity to drive reduction reactions at potentials negative of 0 V_RHE_, consistent with our SEC analysis above (Figure 4).

### 2.6 Photoaccumulated Electrons for Hydrogen Evolution in the Dark

We conclude by exploring the ability of the long‐lived accumulated electrons in MIP‐177(Ti)‐LT to reduce protons to H_2_, exploiting the long lifetime of these electrons to separate the light‐driven generation and dark catalysis processes. It is noteworthy that with careful sealing to prevent oxygen ingress, photoaccumulated electrons in MIP‐177(Ti)‐LT are not fully discharged over 48 h (Figure S9d, Supporting Information). This makes MIP‐177(Ti)‐LT an excellent material for dark photocatalysis under optimized anaerobic conditions, as it accumulates and stores electrons under light irradiation which can later be utilized in the dark.^[^ ^72^ ^]^

**Figure 7:**
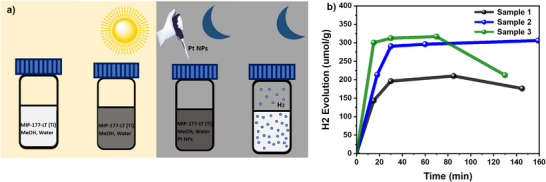
a) Schematic illustration of dark photocatalysis experiments on a MIP‐177(Ti)‐LT suspension: left: steady state of MIP‐177(Ti)‐LT; photoaccumulation of electrons in MIP‐177(Ti)‐LT by pre‐illumination; right: adding Pt co‐catalyst into the system in the dark; hydrogen evolves in the dark. b) Hydrogen evolution in the dark after 30 min (Sample 1 & 2) and 60 min (Sample 3) of 365 nm 200 mW cm^−2^ LED illumination of 3 nominally identical MIP‐177(Ti)‐LT suspension in the presence of methanol (25% by volume). Pt nanoparticle co‐catalyst triggering dark photocatalyis was 2.5 wt.% for Sample 1, and 1.125 wt.% for Sample 2 and 3. Identical results for Sample 2 and 3 confirm photocharging saturation.

We investigated the dark photocatalysis of electron‐saturated MIP‐177(Ti)‐LT after 30 min of LED pre‐illumination (365 nm, intensity of 200 mW cm^−2^) in the presence of methanol, with dark H_2_ production monitored by gas chromatography following the subsequent addition of Pt nanoparticles to the solution in the dark (**Figure** **7**a). A rapid evolution of H_2_ was observed, reaching saturation 30 min after Pt nanoparticles addition (Figure 7b). The total amount of evolved H_2_ reached ≈300 µmol per gram of MIP‐177(Ti)‐LT. Assuming 100% efficiency for electron transfer to the Pt co‐catalyst and for the catalytic production of H_2_, this corresponds to ≈0.6 mmol g^−1^ of electrons (≈600 µmol [e^−^] g^−1^, or ≈58 C g^−1^), which is higher than that reported for a Mn‐Based photochargable MOF.^[^ ^71^ ^]^ Assuming a periodic and defect‐free structure of the material, this corresponds to an electron storage density of 0.1 electrons per Ti atom (27 wt.% of Ti in MIP‐177(Ti)‐LT), or 1.2 electrons per Ti‐oxo cluster on average (see calculations in Section S3.2, Supporting Information). These findings highlight the capacity of MIP‐177(Ti)‐LT for photoaccumulation of reactive electrons, enabling time‐delayed hydrogen evolution with high efficiency.

## 3 Discussion

This study has explored the photophysical properties and charge dynamics of MIP‐177(Ti)‐LT photocatalyst over seventeen orders of magnitude in time (ps to days). TAS studies under pulsed laser excitation demonstrate that MIP‐177(Ti)‐LT exhibits suppressed ultrafast (ps‐ns) charge recombination compared to benchmark MOFs MIL‐125(Ti)‐NH_2_ and UiO‐66(Zr)‐NH_2_ (Figure 2c), leading to the generation of long‐lived electrons with lifetimes extending to the millisecond timescale following pulsed laser excitation (Figure 3c). Optical absorption studies under quasi‐continuous irradiation (20 s) indicate the accumulation of electrons with lifetimes >10 s, whilst studies following prolonged illumination and optimized exclusion of oxygen from the sample resulted in prolonged coloration of the sample assigned to electron accumulation persisting for up to 2 days.

These photoaccumulated electrons are shown to be reactive for both methyl viologen reduction and, following the addition of Pt in the dark post illumination, proton reduction to molecular hydrogen (dark photocatalysis). Following prolonged illumination in the presence of methanol (30 min), the density of accumulated long‐lived electrons is remarkably high, being 1.2 electrons per Ti_12_O_15_ cluster, corresponding to 600 µmol [e^−^] g^−1^. These values are the highest reported for any MOF and are among the highest reported for stable electron storage performance in other materials,^[^ ^77^, ^78^ ^]^ particularly in aqueous systems, as summarized in Table S1 (Supporting Information).^[^ ^71^, ^72^, ^79^, ^80^, ^81^, ^82^, ^83^, ^84^, ^85^, ^86^ ^]^ Our observation of high storage capacity for long‐lived, reactive electron accumulation highlights the photocatalytic and charging properties of MIP‐177(Ti)‐LT.

Among the MOFs discussed above, MIP‐177(Ti)‐LT is the only one which can be visibly photocharged without a hole scavenger (Figure S11a, Supporting Information). This can be attributed to the suppressed recombination of photogenerated charges in this MOF, as indicated by our transient absorption studies under pulsed laser excitation, and the ability of its photogenerated holes to oxidize water (Figures 2c and 3c). We note that although photogenerated charges in MIL‐125(Ti)‐NH_2_ decay quickly without scavengers, MIL‐125(Ti)‐NH_2_ has been reported to accumulate photoexcited species which cause a color change from yellow to blue when in the presence of a hole scavenger and under a N_2_ environment.^[^ ^28^ ^]^ Analogous photoinduced color changes have been reported in TiO_2_ (from white to bluish grey) and various other systems,^[^ ^77^, ^87^, ^88^ ^]^ which in these cases were caused by the accumulation of long‐lived electrons.^[^ ^88^ ^]^ In some cases, these long‐lived electrons have been shown to be reactive enough to drive photosynthetic reactions, as exemplified in carbon nitride systems.^[^ ^79^, ^89^ ^]^

In our studies, without the presence of hole scavenger, MIL‐125‐ NH_2_(Ti) did not exhibit a color change (Figure S11b, Supporting Information) after 1.5 h of 365 nm LED illumination,^[^ ^78^, ^90^ ^]^ indicating that no photocharging occurs without hole scavengers. Similarly, photocharging has been reported for MIL‐125(Ti), with a capacity of 2 electrons per Ti_8_ cluster (equivalent to 0.25 electron per Ti atom) in the presence of a hole scavenger and under illumination intensities three orders of magnitude higher and illumination times 40 times longer than those used for MIP‐177(Ti)‐LT herein.^[^ ^90^ ^]^ Furthermore, MIL‐125(Ti) and MIL‐125(Ti)‐NH_2_ exhibit much lower dark photocatalytic proton reduction rates than MIP‐177(Ti)‐LT, despite the latter being tested under preliminary, non‐optimized conditions.^[^ ^90^ ^]^ Meanwhile, UiO‐66(Zr)‐NH_2_ settled at the bottom of the cuvette (Figure S11c, Supporting Information) under our experimental conditions and did not show any photocharging behavior.

These comparisons underscore the strong electron storage capability of MIP‐177(Ti)‐LT, with potential for further enhancement through further optimisation of the illumination conditions and/or through chemical modification of the MOF structure (i.e., metal ions doping or grafting onto the Ti‐oxo‐cluster.^[^ ^91^, ^92^ ^]^) We suggest that this superior performance is linked to the unique structural features of MIP‐177(Ti)‐LT. Compared with those in UiO‐66(Zr)‐NH_2_ and MIL‐125(Ti)‐NH_2_, the higher nuclearity and closer proximity of the Ti_12_‐oxo clusters in MIP‐177(Ti)‐LT may enhance cluster‐to‐cluster charge separation. Furthermore, the more oxo‐rich coordination environment^[^ ^38^ ^]^ in MIP‐177(Ti)‐LT may aid subsequent charge localisation / stabilisation, further suppressing charge recombination. The persistence of the electrons on MIP‐177(Ti)‐LT further implies that photogenerated holes are effectively extracted and are sufficiently oxidising to drive both methanol and water oxidation, even without electron scavengers. The ability of MIP‐177(Ti)‐LT holes to oxidize water might suggest, though without direct evidence, a possible localisation of the HOMO on the Ti‐O framework,^[^ ^38^ ^]^ while the wide‐bandgap organic linkers may act primarily as structural supports and contribute to the inhomogeneity of the potentials. Notably, MIP‐177(Ti)‐LT sustains its photocharging performance without apparent structural degradation even after repeated photocharging/discharging cycles, consistent with prior reports of its exceptional chemical stability^[^ ^38^, ^39^ ^]^ and its demonstrated cyclability and robustness under photocatalytic conditions.^[^ ^23^, ^40^ ^]^ These findings illustrate the broader potential of structurally tunable MOFs for photocatalysis, where precise control over electronic structure can unlock efficient charge storage and utilisation.

## 4 Conclusion

Our study demonstrates that the Ti_12_ oxocluster carboxylate‐based MOF MIP‐177(Ti)‐LT exhibits notable photoinduced charge accumulation properties compared to other commonly studied MOF photocatalysts. Transient optical studies reveal that, on femtosecond‐second timescales, MIP‐177(Ti)‐LT generates long‐lived electrons solely in water without the need for dedicated hole scavengers. The photoaccumulation of electrons in MIP‐177(Ti)‐LT induces a reversible color change from white to black, lasting over 48 h following 1 h of UV illumination. This long‐lived electron accumulation is correlated with water oxidation to molecular oxygen or alcohol oxidation, and does not degrade the material's structure.

Photoaccumulated electrons in MIP‐177(Ti)‐LT demonstrate high redox reactivity, as evidenced by their rapid reduction of electron acceptors such as molecular oxygen or methyl viologen. Photoaccumulated electrons can also perform time‐delayed photocatalysis, yielding ≈300 µmol g^−1^ hydrogen from water within 30 min following 30 min of pre‐illumination. This corresponds to 600 µmol g^−1^ of stably accumulated electrons in MIP‐177(Ti)‐LT, translating to an average of 0.1 electrons stably accumulated per Ti atom or 1.2 electrons per Ti‐oxo cluster in MIP‐177(Ti)‐LT, highlighting its superior electron storage capability in aqueous conditions compared to previously‐reported photochargeable materials. Future research could focus on optimising MIP‐177(Ti)‐LT for photocatalytic or photocharging applications by refining its structure, understanding charge trapping mechanisms, and evaluating its performance in a wider range of catalytic reactions.

## Conflict of Interest

The authors declare no conflict of interest.

## Author Contributions

S.Y. designed and conducted the experiments, performed data analysis, and wrote the manuscript. K.H. synthesized MIP‐177(Ti)‐LT and UiO‐66‐NH_2_(Zr), and contributed to discussions on MOF structures. S.H. supported SEC measurements, general discussions, and reviewed the draft. F.P. advised on the methyl viologen experiment and reviewed the draft. T.H. supported TAS and PIAS data analysis and participated in general discussions. A.G.‐B., S.N., and H.G. conducted and analysed OER/OWS measurements using GC and EPR. F.P. performed dark photocatalysis measurements, assisted by Y.B. and S.E., for using GC and data analysis. K.D. and N.S. synthesized MIL‐125‐NH_2_(Ti). S.G.‐C., G.M., C.S., and J.D. supervised the project.

## Supporting information



Supporting Information

## Data Availability

The data that support the findings of this study are available from the corresponding author upon reasonable request.
