# Supplementary material for: Photoaccumulation of Long‐Lived Reactive Electrons in a Microporous Ti(IV) Oxocluster‐Based Metal–Organic Framework for Light and Dark Photocatalysis
Source: Adv Mater. 2025 Dec 3;38(8):e17595. doi: 10.1002/adma.202517595 (PMC12878811; doi:10.1002/adma.202517595)
Supplement: Supplementary file 1 — Supporting Information [file ADMA-38-e17595-s001.docx]

Supporting Information

Photoaccumulation of Long-lived Reactive Electrons in a Microporous Ti(IV) Oxocluster Based Metal-organic Framework for Light and Dark Photocatalysis

Shilin Yao,^1^ Katrin Heinzerling,^2^ Sam A. J. Hillman, ^1^ Filip Podjaski, ^*1^ Tianhao He, ^1^ Alberto García-Baldoví, ^3^ Yasmine Baghdadi,^4^ Khaled Dassouki,^5^ Hermenegildo García,^3^ Salvador Eslava, ^4^ Nathalie Steunou,^5^ Soranyel Gonzalez-Carrero,*^1,6^ Sergio Navalón,^7^ Georges Mouchaham,^*2^ Christian Serre,^*2^ and James R. Durrant ^*1^

1. Ms. Shilin Yao, Dr. Sam A. J. Hillman, Dr. Filip Podjaski, Dr. Tianhao He, Dr. Soranyel Gonzalez-Carrero, Prof. James R. Durrant

Department of Chemistry, Imperial College London, Molecular Science Research Hub, White City, London, W12 0BZ, United Kingdom
E-mail: [j.durrant@imperial.ac.uk](mailto:j.durrant@imperial.ac.uk); [soranyel.gonzalez@uv.es](mailto:soranyel.gonzalez@uv.es); [f.podjaski@imperial.ac.uk](mailto:f.podjaski@imperial.ac.uk)

2. Ms. Katrin Heinzerling, Dr. Georges Mouchaham, Dr. Christian Serre
Institut des Matériaux Poreux de Paris, ESPCI Paris, Ecole Normale Supérieure, CNRS, PSL University, 75005 Paris, France

E-mail: [christian.serre@ens.psl.eu](mailto:christian.serre@ens.psl.eupsl.eu); [georges.mouchaham@ens.psl.eu](mailto:georges.mouchaham@ens.psl.eu)

3. Dr. Alberto García-Baldoví, Prof. Hermenegildo García

Instituto de Tecnología Química (CSIC-UPV), Universitat Politècnica de València, Agencia Estatal Consejo Superior de Investigaciones Científicas, Av. de los Naranjos s/n, 46022 Valencia, Spain.

4. Dr. Yasmine Baghdadi, Dr. Salvador Eslava

Department of Chemical Engineering and Centre for Processable Electronics, Imperial College London, London, SW7 2AZ, United Kingdom

5. Mr. Khaled Dassouki, Prof. Nathalie Steunou

Institut Lavoisier de Versailles, Université de Versailles St Quentin en Yvelines, Université Paris Saclay, 45 Avenue des Etats Unis, 78035 Versailles, France.

6. Dr. Soranyel Gonzalez-Carrero

Institute of Molecular Science, University of Valencia, 46980 Paterna, Valencia, Spain

7. Prof. Sergio Navalón

Departamento de Química, Universitat Politècnica de València, Camino de Vera s/n, Valencia

46022, Spain

Contents

[Experimental Procedures 3](#_Toc166067492)

[Supplementary Figures 9](#_Toc166067493)

[Calculations 17](#_Toc166067494)

1. Experimental Procedures

1.1. Spectroscopy

1.1.1. Steady-state Absorption and Emission Spectroscopy

The steady-state UV-Vis absorption spectra were measured using a spectrometer (Cary 5000, Agilent Technologies) in both transmission mode and reflectance mode (240-800 nm), with water in a cuvette used as the reference. The absorbance is therefore obtained by the equation: A=1-T-R, where T stands for transmission and R stands for reflectance. The steady-state emission was measured in the range of 335-500 nm by using a Cary Eclipse Fluorescence Spectrophotometer (Agilent Technologies) upon 320 nm excitation.

1.1.2. Transient Absorption Spectroscopy (TAS)

Transient Absorption Spectroscopy (TAS) is a time-resolved pump-probe technique using a laser as the excitation source. For ultrafast Transient Absorption Spectroscopy (fs-TAS), Sapphire laser system (Solstice) and Helios spectrometers (ultrafast systems) were used. The excitation wavelength is adjustable using an optical parametric amplifier (TOPAS Prime) and frequency mixer (NirUVis). In ultrafast transient absorption spectroscopy system (fs-TAS), covering timescales from femtoseconds to nanoseconds, we excited MIP-177(Ti)-LT at 320 nm (laser intensity: 750 nJ/cm^2^), UiO-66(Zr)-NH_2_ at 355 nm (laser intensity: 200 nJ/cm^2^), and MIL-125(Ti)-NH_2_ at 400 nm (laser intensity: 300 nJ/cm^2^), and probed the photogenerated species in the range of 500-800 nm.

In addition, a homebuilt Diffuse Reflectance Transient Absorption Spectroscopy (DR-TAS) was conducted to investigate charge carrier dynamics over the timescales from microseconds to seconds. This system can be operated in either transmission mode or diffuse reflectance mode. Given the highly scattering nature of MOFs, the MIP-177(Ti)-LT suspension was measured in reflectance mode with a 15% transmittance intensity filter. The MIP-177(Ti)-LT film in water was measured in transmission mode with two 15% transmittance intensity filters. The results obtained from both modes and conditions were found to be equivalent. The laser source was generated by OPOTEK Opolette 355 II with a 7 ns pulse width. Data were recorded using an oscilloscope (Tektronics TDS 2012B) for timescales ranging from microseconds to milliseconds, and a DAQ (National Instruments, NI USB-6211) for timescales from milliseconds to seconds. The probe light was generated from a 100 W tungsten lamp (Bentham IL1) with power supplied a Bentham 605 power supply. The samples were excited at 355 nm with laser intensity of 280 µJ/cm^2^, and a laser frequency of 1 Hz. The probed wavelengths range from 500 to 1000 nm, with an average measurement number of 40.

1.1.3. Photoinduced Absorption Spectroscopy (PIAS)

Photoinduced Absorption (PIAS) Spectroscopy closely resembles TAS but distinguish itself by employing LED light pulses with durations extending to several seconds as pump light source, in contrast to the high-intensity pulsed lasers used in TAS. While TAS is employed to probe a sample under transient conditions, PIAS, on the other hand, finds its application in the study of photocatalysts under quasi-steady state conditions. During these longer irradiation periods, photogenerated charges can accumulate. The LED used in this study had a wavelength of 365 nm.

1.1.4. Spectroelectrochemistry (SEC)

A three-electrode cell was built in a 1 cm cuvette containing a Pt mesh counter electrode, Ag/AgCl as reference electrode, and MIP-177(Ti)-LT film deposited on fluorine-doped tin oxide (FTO, Pilkington) as working electrode. The electrolyte was 0.1 mol/L Na_2_SO_4_ water solution, with Na_2_SO_4_ to increase conductivity, degassed with N_2_. SEC absorption difference spectra were acquired using an in-house setup operating in transmittance mode (Schematic shown in Fig. S5b). The probe beam was generated using a 10 mW tungsten-halogen lamp (Thorlabs SLS201L/M with colour-temperature balancing filter FGT05165 and collimation package SLS201C attached), focused onto the sample (in the three-electrode cell) and then directed into a liquid light guide (Thorlabs LLG5-4Z, 5 mm diameter, 420-2000 nm) connected to a Maya2000Pro spectrometer (OceanOptics). A 410 nm long-pass filter was placed before the sample to remove high-energy UV/blue light, preventing photochemical excitation and improving signal stability. The applied potential was increased in steps of 100 mV vs the Ag/AgCl reference electrode (using a PGSTAT101 potentiostat (Metrohm Autolab) and a transmittance spectrum (500-900 nm) was acquired at each applied potential. Data acquisition was managed using homebuilt LabView software (https://opensourcespectroscopy.com/sec_code/). Five consecutive spectra (100 ms per spectrum) were acquired at each potential and averaged together. At each potential step, a waiting time of 0.1 seconds is applied prior to the spectrum being measured. Absorbance difference spectra were calculated versus a transmittance spectrum taken at 0 V vs Ag/AgCl. The conversion of voltage from Ag/AgCl to RHE is described in Section 3.4.

1.1.5. Electron Paramagnetic Resonance Spectroscopy (EPR)

EPR spectra were recorded on a Bruker EMX-12 instrument at 100 K operating in X band at 9.433 GHz, modulation amplitude of 1 G and modulation frequency of 100 kHz. Before measuring the EPR spectrum of MIP-177(Ti)-LT, a background signal was recorded using the EPR quartz tube. Then, the solid (10 mg) was placed in an EPR quartz tube (4 mm diameter) and purged with argon to remove oxygen. EPR measurements were performed under dark and under simulated sunlight irradiation (AM 1.5 filter; 100 mW/cm^2^).

1.1.6. Gas Chromatography (GC)

For oxygen evolution measurements, gas-phase products were analysed using an Agilent 490 MicroGC system equipped with two µGC compact columns: a Molecular Sieve 5Å column operating at 60 °C and a PoraPLOT Q column operating at 70°C) and a thermal conductivity detector (TCD). Argon was used as carrier gas.

For dark photocatalytic hydrogen evolution measurements, gas chromatography (GC) analysis was performed using a Shimadzu QP 2030NX gas chromatograph equipped with a barrier ionization discharge (BID) detector, which is suitable for the detection of a wide range of volatile compounds. An SH-Rt-Q-BOND column was used for separation, with the oven operated isothermally at 60 °C. This setup enabled efficient analysis of permanent gases and light hydrocarbons under stable temperature conditions.

1.1.7. Gas Chromatography Coupled to a Mass Spectrometer (GC-MS)

MIP-177(Ti)-LT (1 mg) was dispersed into labelled H_2_^18^O (500 μL) using a quartz reactor (10 mL). The system was sonicated for 20 min at 500 W to ensure a good suspension. Subsequently, the reactor was purged with argon to remove oxygen from the system. The reactor was then irradiated with simulated sunlight irradiation (100 mW/cm^2^) from a Hamamatsu LC8-150 W Xe lamp equipped with an AM 1.5 filter. The gaseous products were analysed using an Agilent 8890 gas chromatography system coupled with a 5977C mass-selective detector (GC/MSD). The GC is equipped with a HP-5/ MS-UI Column (30 cm, 0.250 mm-0.5µm).

1.2. Material Synthesis

MIP-177(Ti)-LT (MIP: MIP stands for Materials from Institute of Porous Materials of Paris) was prepared by mixing Ti(*i*PrO)_4_ and di(isophthalyl)methane (H_4_-mdip) in formic acid under reflux conditions during 3 days. The solid was the collected by centrifugation and thoroughly washed by ethanol under reflux to remove the residual free linkers. UiO-66(Zr)-NH_2_ was prepared by reacting 2-aminoterephthalic acid (BDC-NH_2_) (2.89g, 16 mmol), HCl 36.5-38.0%, (5.4 mL) and zirconium(IV) (acac)_2_ (7.8g, 16 mmol) in 32 mL of ethanol under reflux for up to 72h In a 100 mL round bottom flask. The resulting solid was then recovered by centrifugation and wash 3 times with 50 mL of ethanol and vacuum dried in oven. Both materials were synthesised by Katrin Heinzerling (Institut des Matériaux Poreux de Paris, France) and have a particle size of approximately 100-400 nm.

MIL-125(Ti)-NH_2_ was synthesised by Khaled Dassouki (University Versailles St Quentin en Yvelines, France) with a particle size in the nanoscale, as previously reported ^[80]^. Briefly, 4.125 g of 2-aminoterephtalic acid were dissolved at room temperature in a mixture of 60 ml of DMF (N,N’-Dimethylformamide) and 75 mL of methanol in a 250 ml round-bottom flask. The mixture was heated to 100 ^o^C under stirring until complete dissolution of the ligand. Then, 4.5 mL of titanium isopropoxide and 0.3 mL of water were added. The resulting yellow suspension was refluxed at 100 ^o^C for 96 hours. The obtained yellow solid was recovered by centrifugation (13000 rpm, 10 min), washed with 60 mL of DMF to remove all excess ligands followed by 80 mL of hot absolute ethanol to remove residual DMF.

1.3. Sample Preparation for Spectroscopy

Standard suspensions of MOFs were prepared by dispersing 3.2 mg MOFs in 500 µL water (6.4 mg/mL) and sonicated for 10 minutes, resulting in a well-dispersed suspension. Subsequently, 300 µL of this suspension was added to 2 mm quartz cuvette (Hellma, 110-2-40), sealed with rubber septum and parafilm, and degassed with argon for 15 minutes. For steady-state absorption and emission measurement, 10 µL of MOF suspension were added in 500 µL water (0.1 mg/mL).

The MOF films were prepared by drop-casting 150 µL of the previously described standard suspension onto a clean glass substrate measuring 1.5 x 1 cm, allowing the solvent to evaporate at room temperature. The film was immersed in a 20 mL quartz cuvette with optical path of 2 cm, containing 8 mL of water, carefully sealed, and degassed with argon for 15 minutes. For SEC measurement, 50 µL of MOF suspension is drop-casted onto a clean FTO substrate measuring 1 x 1 cm and evaporate at room temperature.

1.4. Oxygen Evolution Measurement

1.4.1. Gas Chromatography

A total of 10 mg of MIP-177-LT was dispersed in 20 mL of a 1.5 mol/L (NH₄)₂Ce(NO₃)₆ solution inside a 51 mL quartz reactor. The mixture was sonicated for 20 min using a Bransonic CPX5800E ultrasonic bath to ensure a uniform suspension of the catalyst. To remove residual O₂, a degassing and Ar purging cycle was performed three times. The reactor was then irradiated with a 150 W Xe lamp (*Hamamatsu-Lightningcure LC8)* equipped with an AM 1.5 cut-off filter and an intensity of 100 mW/cm^2^. The gaseous products were analysed and quantified using an Agilent 490 Micro GC system equipped with a Mol*S*ieve 5Å column, with Ar as the carrier gas. Reactor temperature and pressure were carefully controlled to ensure precise quantification*.*

1.4.2. Clark Electrode

To investigate the role of photogenerated holes in MIP-177(Ti)-LT, an in situ photocatalytic water oxidation for oxygen evolution experiment was designed to determine whether the photogenerated holes in MIP-177(Ti)-LT can oxidise water in the absence of hole scavenger. Since oxygen can react with the photogenerated electrons in MIP-177(Ti)-LT very quickly, an electron scavenger, Na_2_S_2_O_8_, was added to the system. A mixture of 8 mg of MIP-177(Ti)-LT and 95.2 mg Na_2_S_2_O_8_ (0.1 mol/L) were dissolved/dispersed in 4 mL deionised water and sonicated for 10 minutes to ensure a good suspension. The suspension was then transferred into a 10 mm light path quartz cuvette with spiral cap and degassed with Ar for 30 minutes. The system was illuminated with 365 nm LED with intensity of 100 mW/cm^2^ and an illumination area of 1.5 cm^2^, while measuring in situ oxygen evolution using a Clark electrode (Unisense), while stirring.

1.5. Overall Water Splitting

Photocatalytic overall water splitting (OWS) experiments were conducted following the same procedure described in Section 1.4.1 for the oxygen evolution reaction, except without the use of (NH_4_)_2_Ce(NO_3_)_6_ as hole scavengers or Pt co-catalyst. A total of 10 mg of MIP-177-LT was dispersed in 20 mL of deionized water inside a 51 mL quartz reactor. The suspension was sonicated for 20 min in a Bransonic CPX5800E ultrasonic bath to ensure a homogeneous dispersion of the photocatalyst. To remove residual dissolved oxygen, a degassing and Ar purging cycle was performed three times. The reactor was then irradiated using a 150 W Xe lamp (Hamamatsu-Lightningcure LC8) equipped with an AM 1.5 cut-off filter and an intensity of 100 mW/cm^2^. The evolved H_2_ and O_2_ gases were analysed and quantified using an Agilent 490 Micro GC system equipped with a MolSieve 5Å column, employing Ar as the carrier gas. Reactor temperature and pressure were continuously monitored and maintained to ensure accurate gas quantification throughout the experiment.

1.6. Dark Photocatalysis

To test hydrogen evolution after illumination (dark photocatalysis), a suspension containing 8 mg of MIP-177(Ti)-LT, 3 mL of deionised water and 1 mL of methanol was inserted in a 9.1 mL glass vial equipped with a septum. It was sonicated for 10 min and degas for 40 min while stirring. Sample 1 & Sample 2 were pre-illuminated by a 365nm LED (200W/cm²) while stirring for 30 min to charge, and Sample 3 was pre-illuminated for 60min. After illumination, the sample vial was wrapped with Al foil to protect from further illumination, and Pt nanoparticles, were added into the system to trigger dark photocatalysis: 200 µl of Pt nanoparticles (2.5 wt%) to Sample 1, and 100µl (1.125 wt%) each for Sample 2 and 3. Hydrogen evolution was monitored using Gas Chromatography (GC-2023, Shimadzu). A gas-tight needle was inserted through the septum, and 100 μL of gas was withdrawn from the headspace and immediately injected into the GC. The sample extraction is assumed to be minimally invasive considering an approximate headspace of 5 mL. Testing was stopped when hydrogen concentration did not increase further.

2. Supplementary Figures


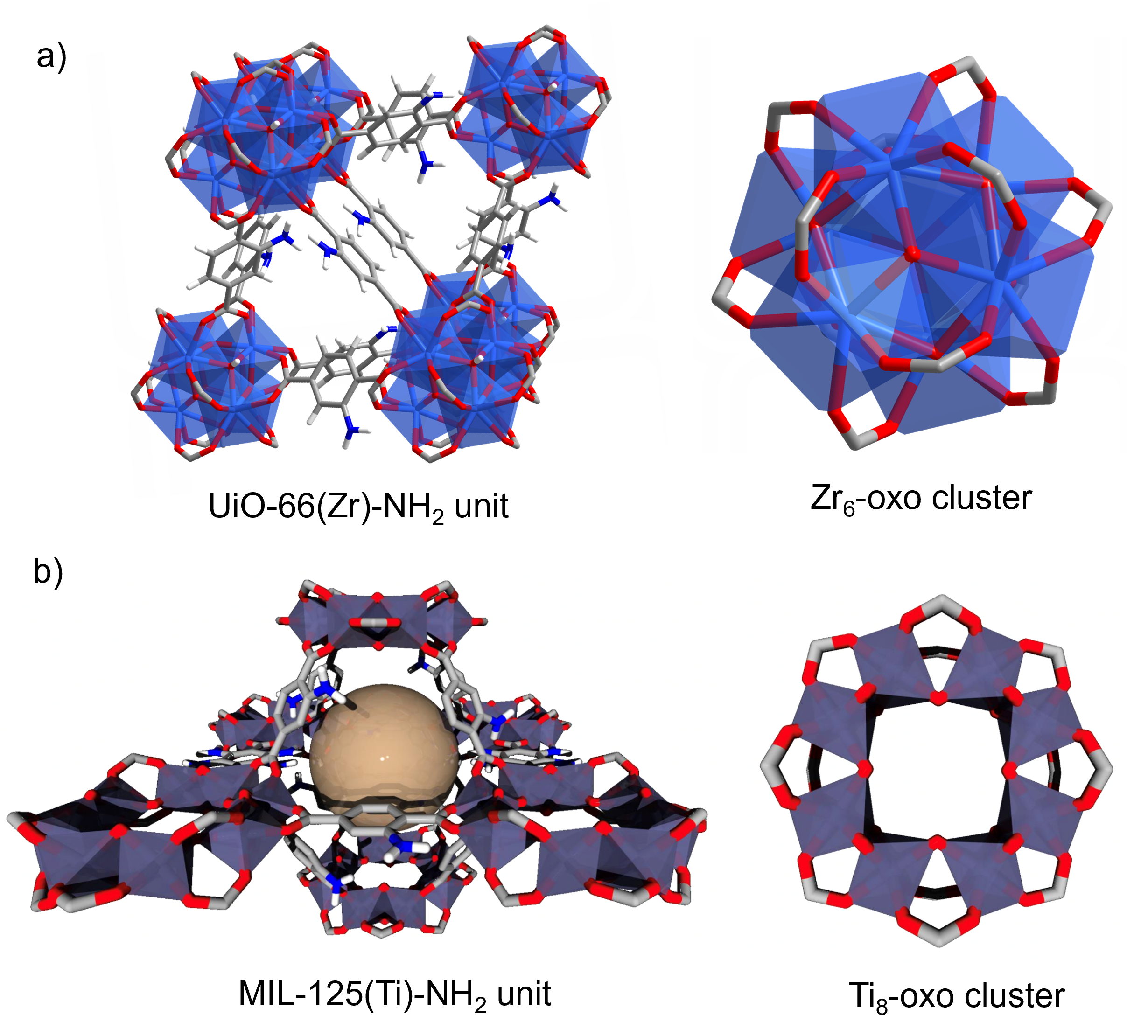


**Figure S1.** Crystal structures of (a) UiO-66(Zr)-NH_2_ (colour codes: ZrO_7_/_8_, royal blue polyhedra; C, grey; O, red; N, blue; H, white) and (b) MIL-125(Ti)-NH_2_ (colour codes: Ti, dark blue; Zr, royal blue; C, grey; N, blue; O, red; H atoms omitted for clarity; the van der Waals sphere indicates the cavity, representing the octahedral cage). In both cases, the frameworks and the inorganic building units are shown on the left and right sides, respectively.


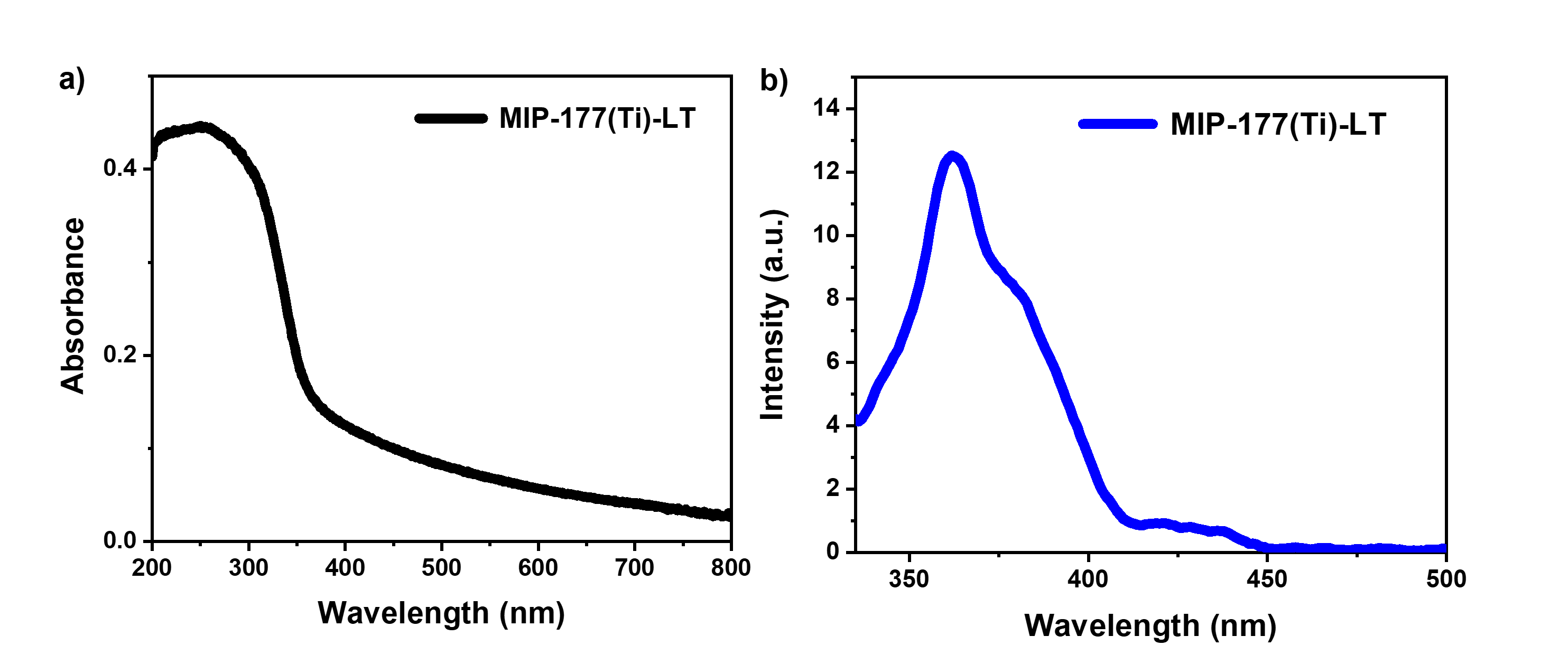


**Figure S2.** a) Unnormalised steady-state UV-Vis absorbance of 0.1 mg/mL MIP-177(Ti)-LT water suspension measured in 2 mm cuvette, b) Unnormalised emission spectrum (blue, λex=320 nm) of 0.1 mg/mL MIP-177(Ti)-LT water suspension measured in 1 cm PL cuvette.


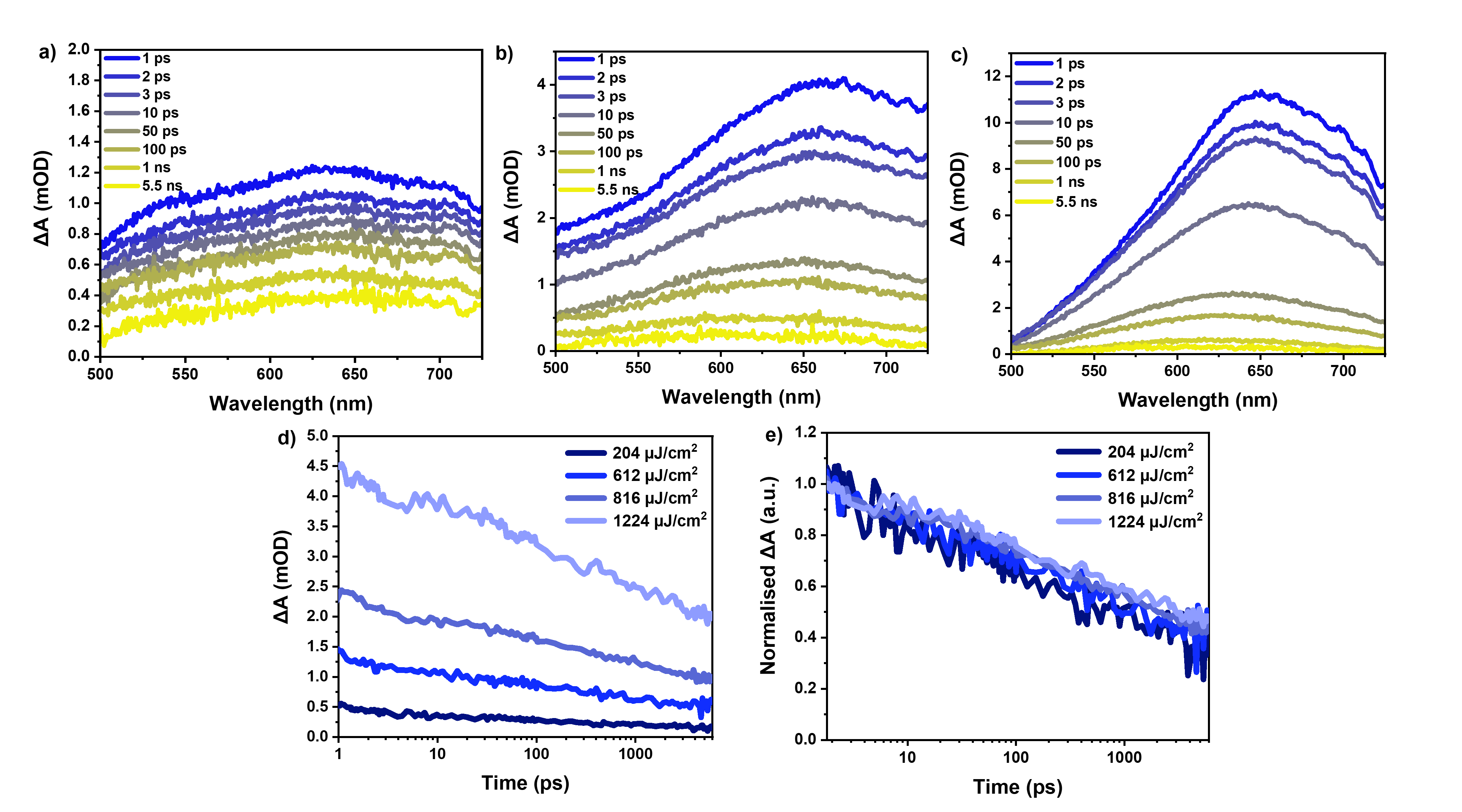


**Figure S3.** fs-TAS spectrum of a) MIP-177(Ti)-LT b) MIL-125(Ti)-NH_2_ c) UiO-66(Zr)-NH_2_ measured on film in water, excitation wavelength of 320 nm pulsed laser (intensity: 640 uJ/cm^2^, frequency: 500 Hz). d) Unnormalised and e) Normalised fs-TAS kinetics (log scale) of MIP-177(Ti)-LT film in water probed at 700 nm, excitation wavelength of 320 nm, pulsed laser with intensity ranges from 204 µJ/cm^2^ to 1224 µJ/cm^2^.


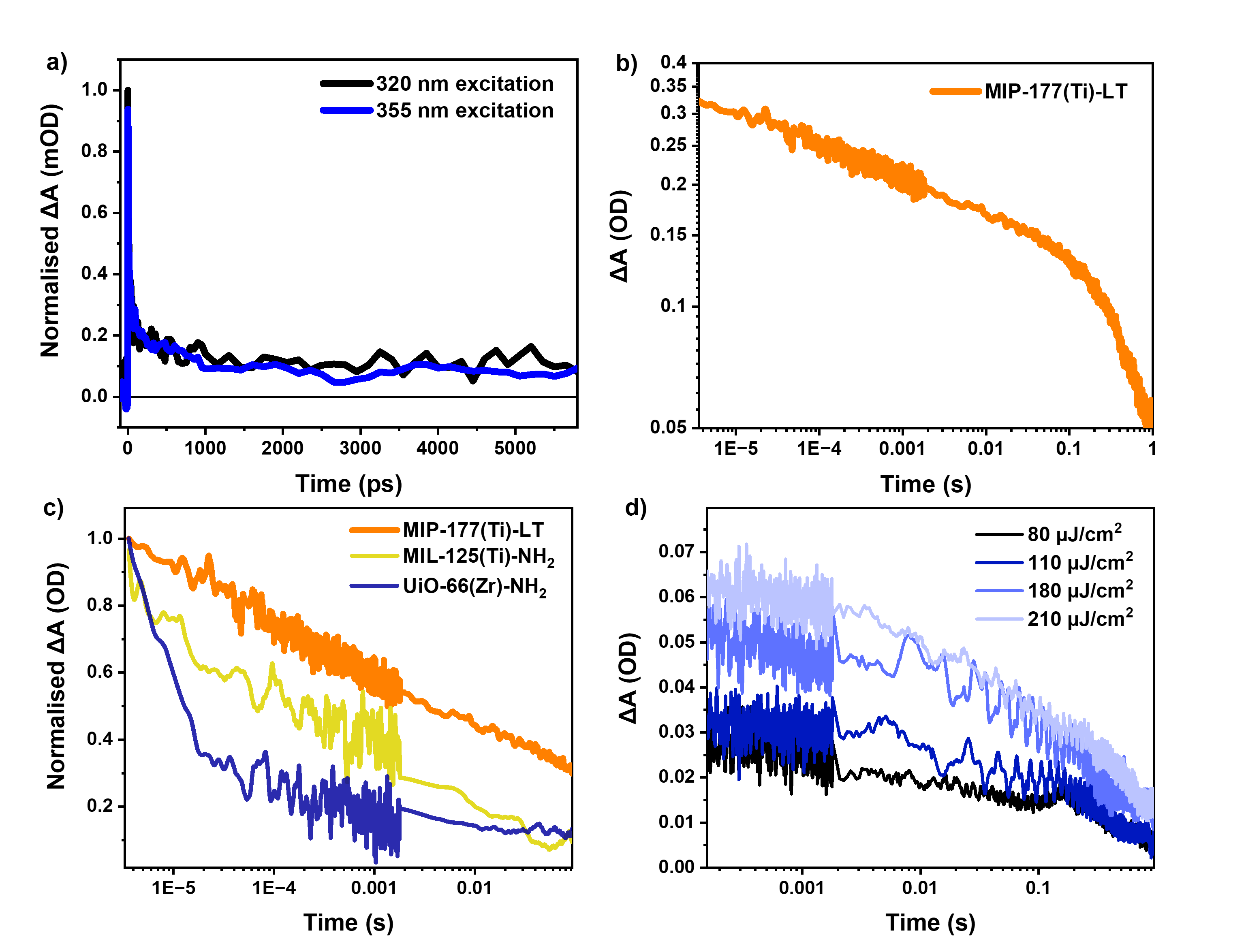


**Figure S4.** a) Normalised fs-TAS kinetics of the MIP-177(Ti)-LT film in water, probed at 650 nm and excited by 320 nm and 355 nm laser with an intensity of 400 µJ/cm^2^. b) DR-TAS kinetics of MIP-177(Ti)-LT film in water, excited by 355 nm pulsed laser (intensity: 280 µJ/cm^2^, frequency: 1 Hz), probed at 650 nm in log-log scale. c) Normalised DR-TAS kinetics of MIP-177(Ti)-LT, MIL-125(Ti)-NH_2_ , and UiO-66(Zr)-NH_2_ water suspension (6.4 mg/mL)), probed at 650 nm (excitation wavelength: 355 nm, intensity: 280 uJ/cm^2^, frequency: 1 Hz). d) DR-TAS kinetics of MIP-177(Ti)-LT water suspension (3.2 mg/mL) with intensity range from 80 µJ/cm^2^ to 210 µJ/cm^2^.


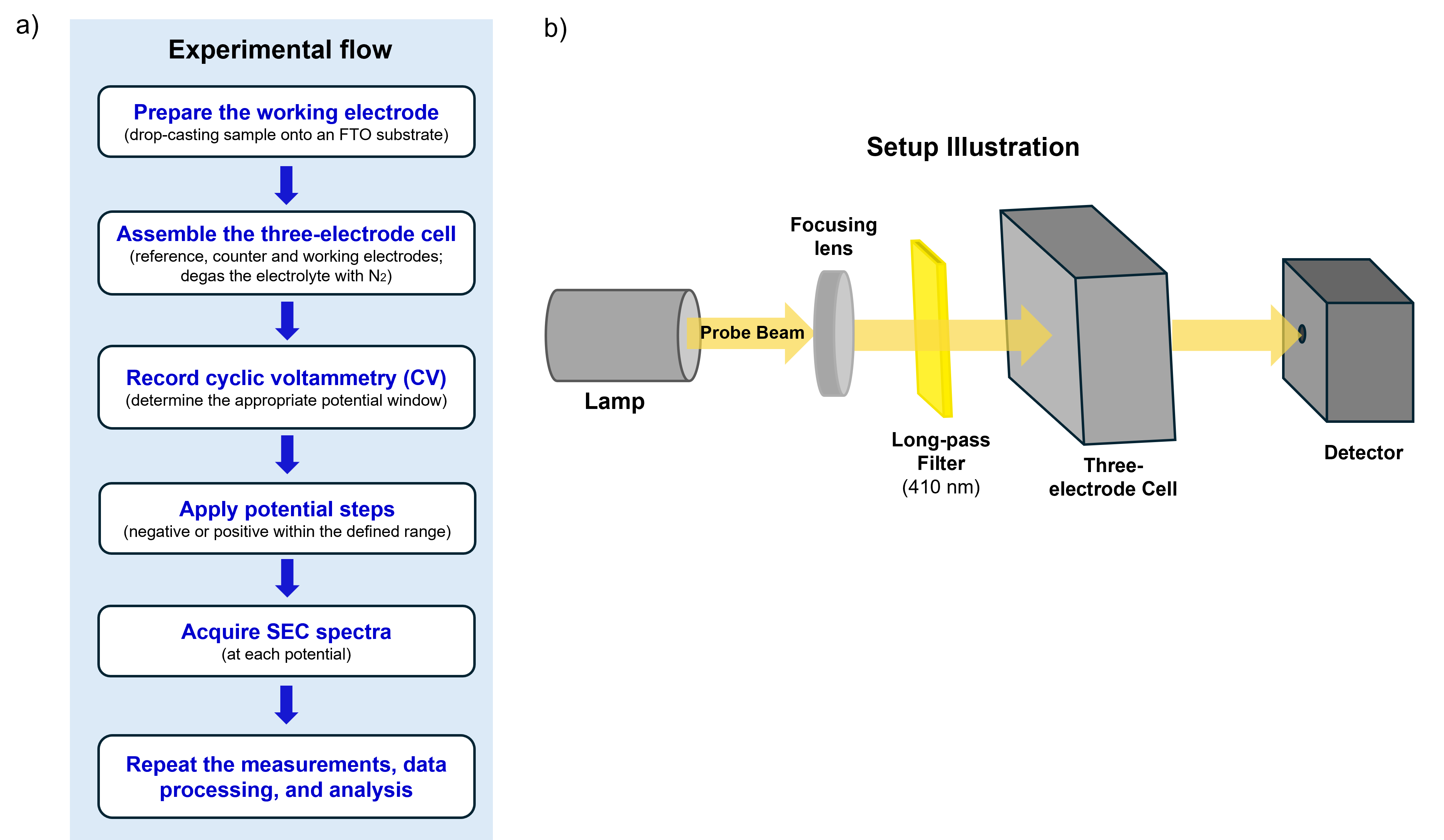


**Figure S5**. Schematics of experimental flow and setup for spectroelectrochemistry measurements (see detailed description in SI Section 1.1.4.). a) Experimental workflow illustrating the stepwise procedure for SEC data acquisition, including electrode preparation, cell assembly, cyclic voltammetry for potential range determination, potential stepping, and spectral recording. b) Simplified optical-electrochemical setup showing the arrangement of the light source (Thorlabs tungsten-halogen lamp), focusing lens, 410 nm long-pass filter, three-electrode cuvette cell, and OceanOptics detector aligned along the probe beam path.


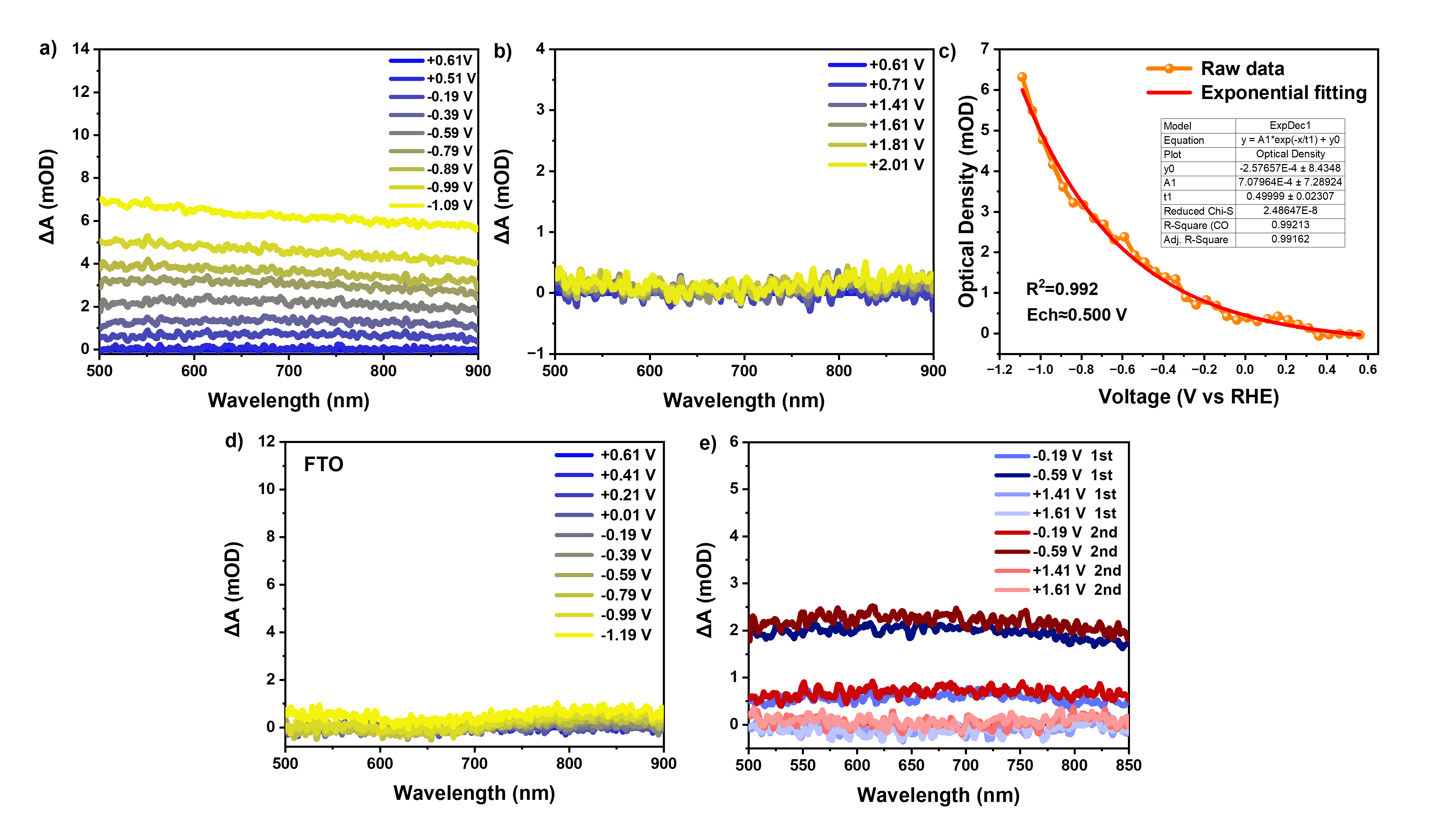


Figure S6. Spectroelectrochemistry absorbance difference spectra of MIP-177(Ti)-LT measured on FTO film in water (degassed with N_2_) with a) increasingly more negative applied potential, from +0.61 V to -1.09 V vs RHE and b) increasingly positive applied potential, from +0.61 V to +2.01 V vs RHE, using Ag/AgCl as reference electrode. c) Fitted potential-dependent absorption of MIP-177(Ti)-LT at 650 nm, measured on FTO film in 0.1 mol/L Na_2_SO_4_ aqueous solution with applied potential, from +0.61 V to -1.09 V vs RHE. d) Background spectra of pure FTO under negative potentials (vs Ag/AgCl reference electrode) from +0.51 V to -1.19 V vs. RHE. e) Reversibility test: spectra measured from negative potentials (-0.19 V to -0.59 V) to positive potentials (+1.41 V and +1.61 V) vs. RHE, then repeating the cycle. The results demonstrate good reversibility. All measurements were conducted in a pre-degassed (N₂-purged) aqueous environment.

**
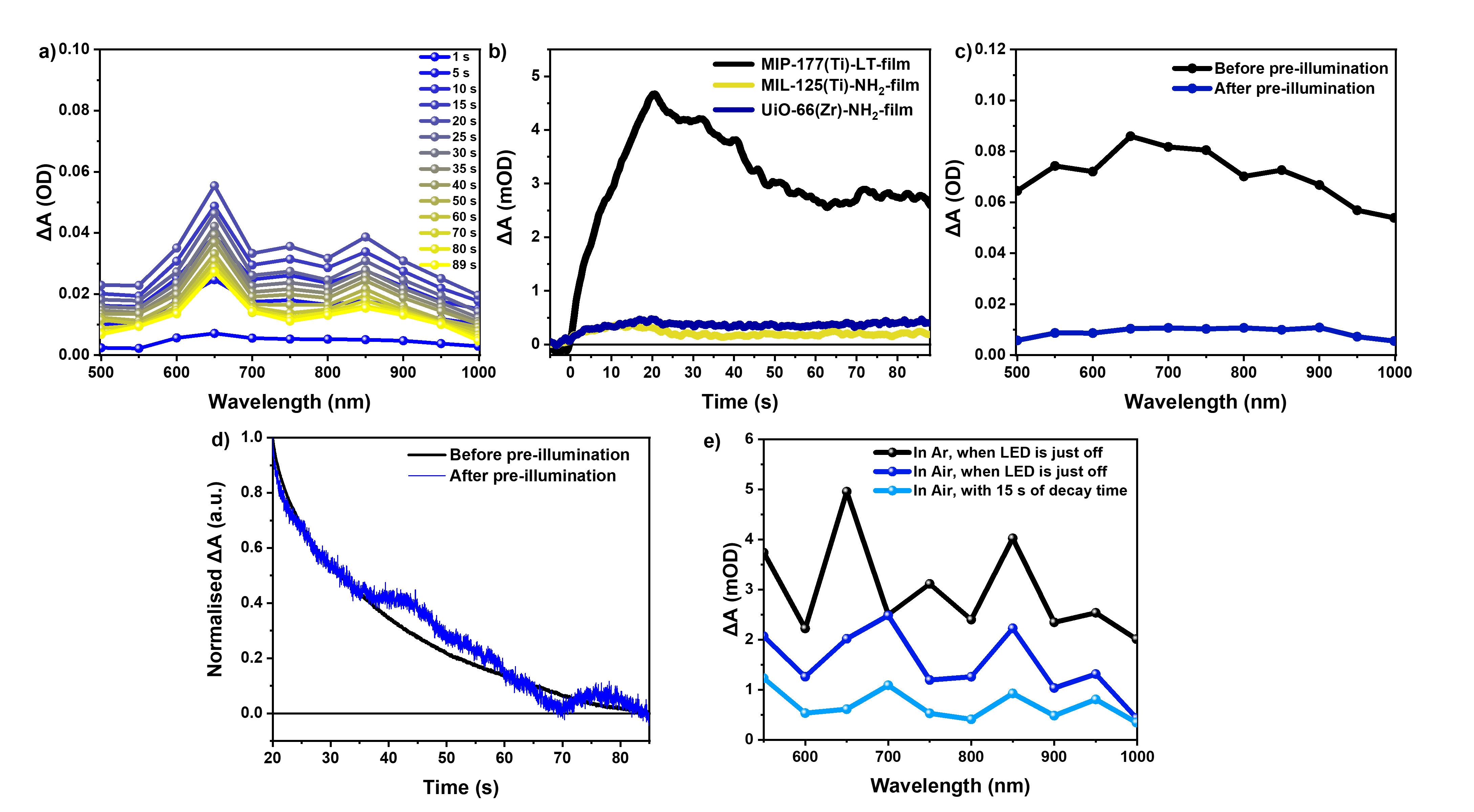
**

**Figure S7.** a) PIAS spectra of MIP-177(Ti)-LT in suspension with 20 seconds LED on and 70 seconds of LED off. b) PIAS kinetics comparison of MIP-177(Ti)-LT, MIL-125(Ti)-NH_2_, and UiO-66(Zr)-NH_2_ thin films in water, degassed with Ar, excited by 365 nm LED (13.64 mW/cm^2^), probed at 650 nm, and measured in reflectance. c) Comparison of the PIAS spectra of MIP-177(Ti)-LT suspension at 20 seconds before and after one hour of pre-illumination. d) Normalised (at 20 s) kinetics of MIP-177(Ti)-LT suspension before (black line) and after (blue line) 1 h of continuous LED pre-illumination (365 nm, 13.64 mW/cm^2^) probed at 650 nm. e) PIAS spectra of MIP-177(Ti)-LT thin film at 20 seconds when LED is just off, in the condition of Ar (black) and air (bright blue), and PIAS spectrum of MIP-177(Ti)-LT with 15 s decay time (light blue). LED excitation wavelength: 365 nm, intensity: 13.64 mW/cm^2^.


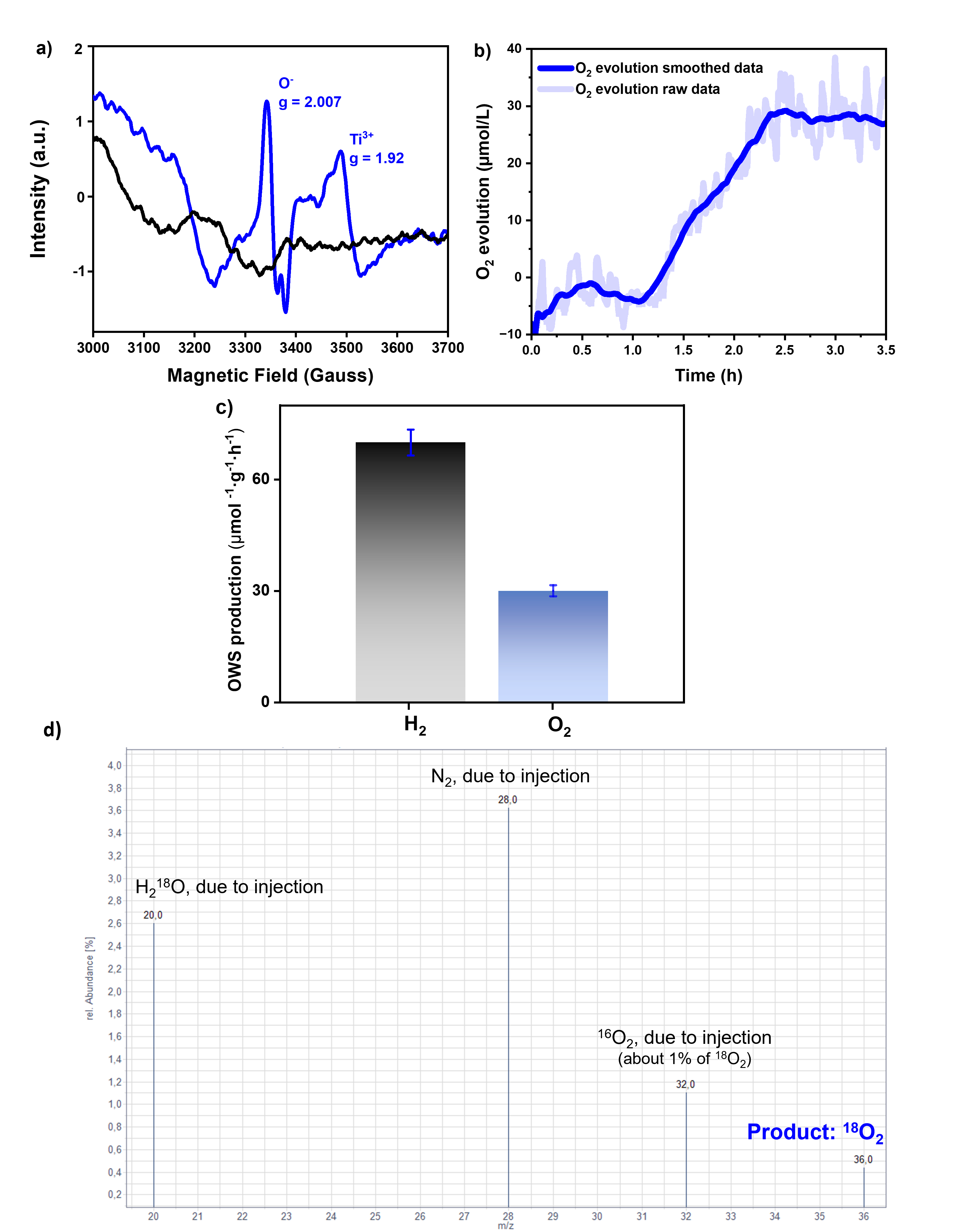


**Figure S8**. a) EPR spectra of MIP-177-LT(Ti) recorded under dark (black line) or simulated sunlight irradiation (blue line) collected at 100 K. b) *In situ* measurement of photocatalytic oxygen evolution using a Clark electrode. Reaction conditions: MIP-177-LT(Ti) (8 mg), H_2_O (4 mL), electron scavenger Na_2_S_2_O_8_ (0.1 mol/L), 365 nm LED illumination (100 mW/cm^2^, light on throughout the measurement). c) Photocatalytic overall water splitting using MIP-177-LT(Ti) under simulated sunlight irradiation. Reaction conditions: MIP-177-LT(Ti) (10 mg), H_2_O (20 mL), simulated sunlight illumination (100 mW/cm^2^). d) GC-MS obtained after 200 μL gas injection of reactor headspace after photocatalytic overall water splitting reaction (2 h) using H_2_^18^O (1 mL) and MIP-177-LT(Ti) (10 mg) under simulated sunlight irradiation. The observed m/z peaks at 32 and 28 were associated with the presence of ^16^O_2_ and N_2_ from air together with H_2_^18^O, respectively, that appeared at the same retention time than ^18^O_2_ and were not separated by our chromatographic column (Agilent HP-5MS).


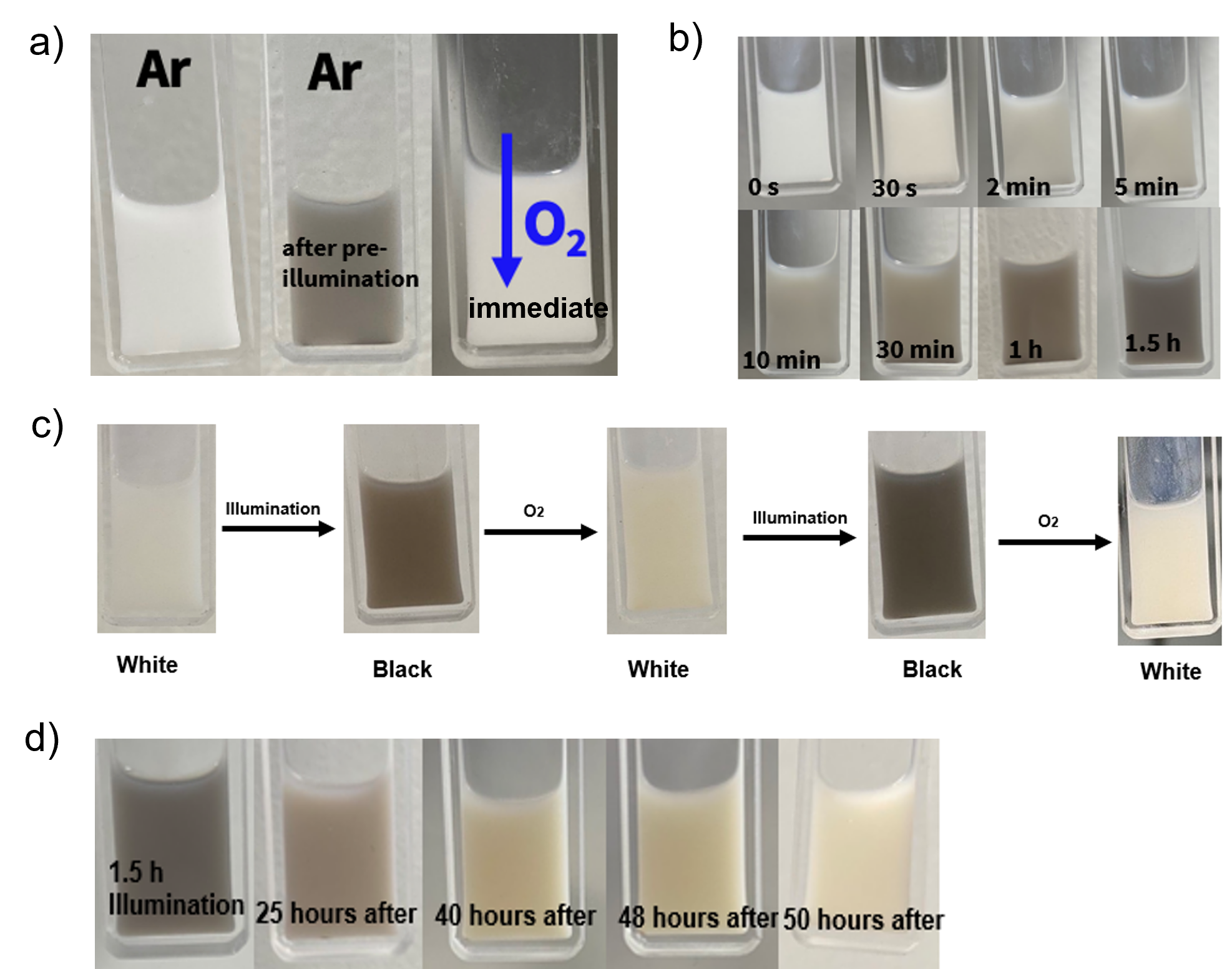


**Figure S9.** Photos depicting a) the immediate reactivity of photoaccumulated electrons in MIP-177(Ti)-LT with O_2_ b) the slower electron accumulation without methonal, suggested by the slower rate of colour change after 0s, 30 s, 2 min, 5 min, 10 min, 30 min, 1h and 1.5 h of pre-illumination c) reversibility of photocharging and discharging in MIP-177(Ti)-LT under pre-illumination or exposure to O_2_. The ground state of MIP-177(Ti)-LT is white; upon 365 nm LED illumination (13.6 mW/cm²), the material turns black. Immediate O₂ injection restores the white colour. After degassing with Ar, re-illumination turns the material black again, while subsequent O₂ injection reverses the colour change. This process can be repeated cyclically d) the discharging process occurred in the dark in MIP-177(Ti)-LT without methanol and under 1.5 h of pre-illumination.


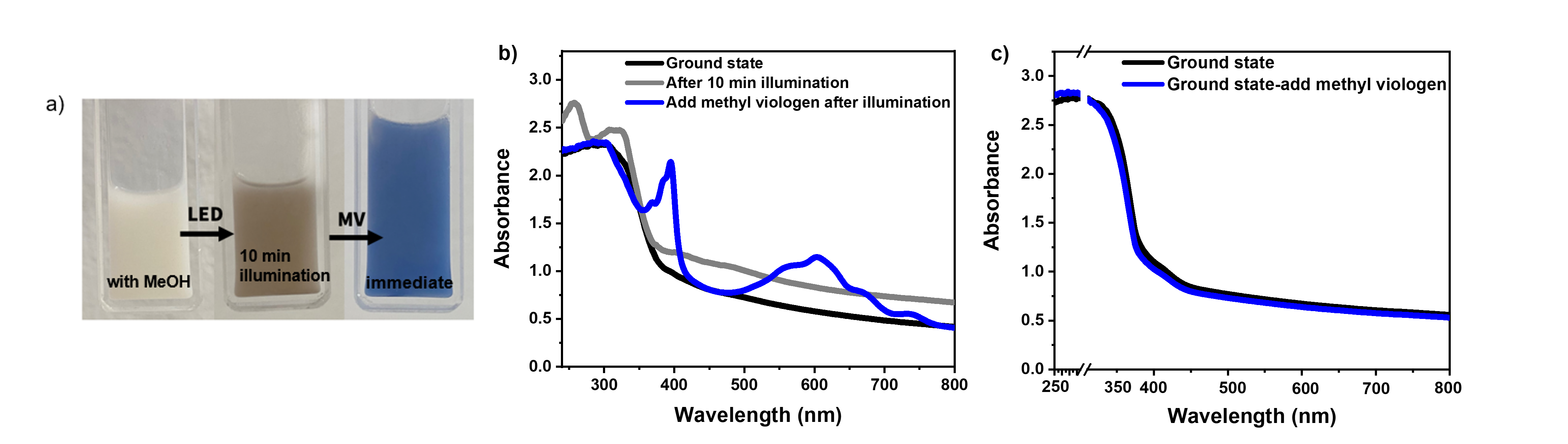


**Figure S10.** a) Photos depicting immediate colour change from black to blue after adding methyl viologen, in the presence of methanol. b) UV-Vis absorption spectra of MIP-177(Ti)-LT in the presence of methanol (black), after 10 mins of pre-illumination (grey), and after adding methyl viologen (blue). c) UV-Vis absorption spectra of MIP-177(Ti)-LT without pre-illumination in the presence of methanol (black) and after adding methyl viologen (blue).


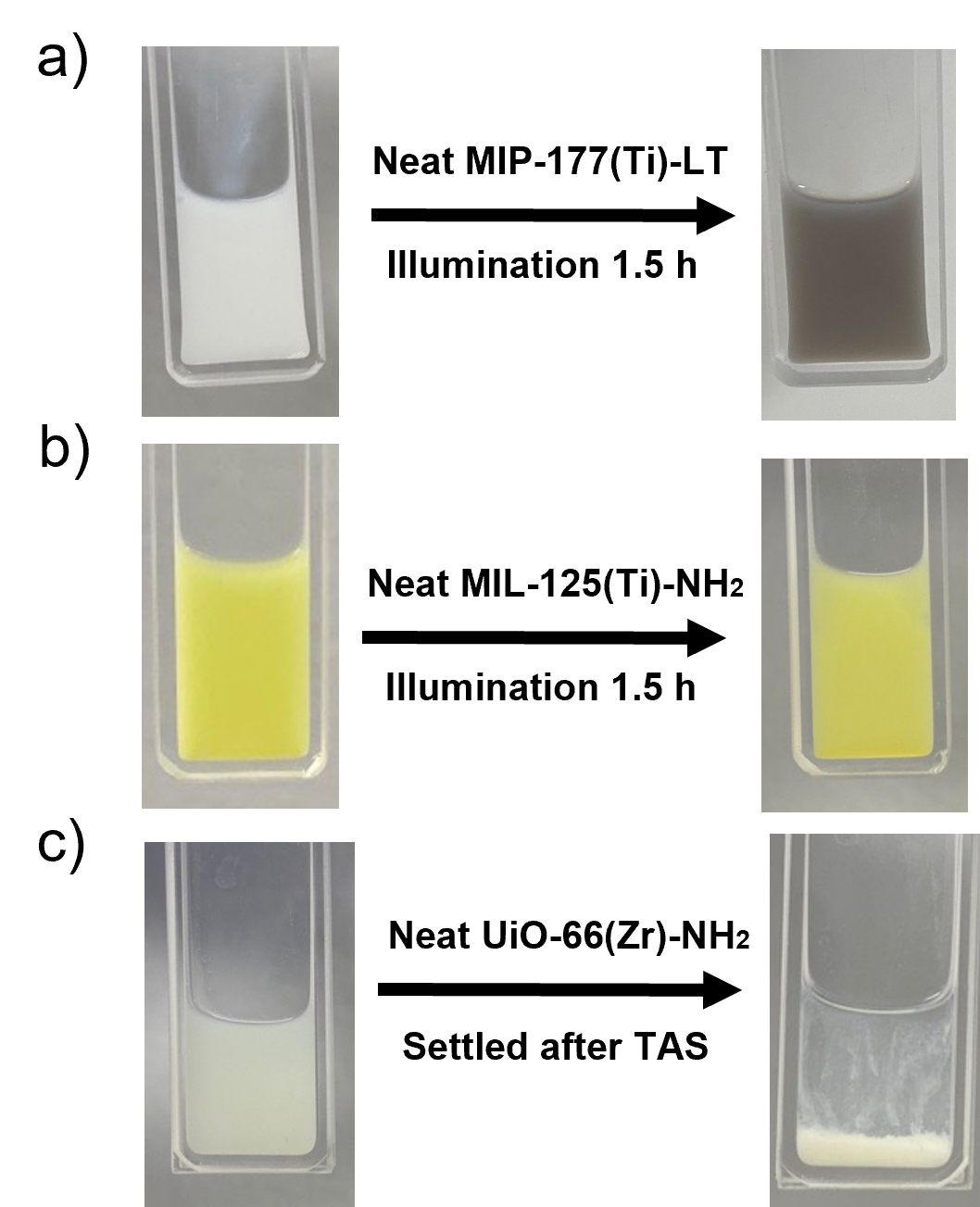


**Figure S11.** Photos depicting the photocharging ability in MOF suspensions in water under the same illumination conditions: a) photoinduced colour change in MIP-177(Ti)-LT due to electron accumulation; b) MIL-125(Ti)-NH_2_ is unable to be photocharged in the absence hole scavenger; c) UiO-66(Zr)-NH_2_ settled to the bottom after TAS measurement and did not exhibit photocharging behaviour.

**Table S1.** Comparison of photogenerated electron storage capacities for various materials. The values are presented as the maximum capacity of stored electrons in micromoles per gram (µmol [e^-^]∙g^-1^), as reported in recent literature ^[68,69,76–83]^.

| Material | Electron capacity  µmol [e^-^]∙g^-1^ | Experimental conditions | Comment | Reference |
| --- | --- | --- | --- | --- |
| Carbon Nitride K-PHI = NCN-CNx | 354 | Aqueous, alcohol donor (4-MBA), Ar purged | N/A | 69 |
| Carbon Nitride K-PHI | 701 | Non-aqueous (MeCN), Benzylamine donor, Ar purged | N/A | 76 |
| Carbon Nitride K-PHI | 957 | Non-aqueous (MeCN), benzylamine donor, CO_2_ purged | N/A | 76 |
| Carbon Nitride mpg-CNx | 43 | Non-aqueous (MeCN), Benzylamine donor, CO_2_ purged | N/A | 76 |
| Carbon Nitride Mel-PHI | 30 | Aqueous, alcohol donor (TEOA), Ar purged | N/A | 77 |
| Carbon Nitride Na/K-PHI | 32 | Aqueous, alcohol donor (methanol), Ar purged | N/A | 77 |
| Carbon Nitride CN-NH-CN | 1460 | Non-aqueous (MeCN), DIEPA donor, N_2_ purged | N/A | 78 |
| MOF MIL-125 (Ti) | 65 | Aqueous, alcohol donor (TEOA), N_2_ purged | N/A | 79 |
| MOF MIL-125(Ti)-NH_2_ | 146 | Aqueous, alcohol donor (TEOA), N_2_ purged | N/A | 79 |
| MOF-253 (Al) | 155 | Non-aqueous (MeCN), alcohol donor (TEOA), Ar purged | N/A | 80 |
| MOF MnBr-253 (Al) | 430 | Non-aqueous (MeCN), triethylamine donor, Ar purged | Not cyclable; material loses most capacity after first use | 68 |
| **MOF MIP-177(Ti)-LT** | 600 | Aqueous, alcoloh donor (MeOH), N_2_ purged | N/A | This work |
| COF 'NDI-COF' | 1400 | Aqueous, alcohol donor (4-MBA), Ar purged | Electrical quantification only; no dark reaction shown-accumulated energy possibly not reductive enough for HER | 81 |
| Transition metal oxide NbWO_6_ | 14 | Aqueous, alcohol donor (MeOH), Ar purged | N/A | 82 |
| TiO_2_ based nanoparticles | 2169 | Non-aqueous (toluene), likely alcohol donor (ethanol), Ar purged | Titrated with 2,4,6-tri-tert-butylphenoxyl or TEMPO; alcohol donor is not always clearly specified; toluene-based photocharging excluded. | 83 |

3. Calculations

3.1. Photoaccumulated Electrons in Methyl Viologen Experiment

According to the Beer-lambert law, $A=\epsilon\boldsymbol{\cdot}c \cdot l$, where $A$ is absorbance, *ϵ* is extinction coefficient, *c* is the concentration, and $l$ is the light path length.

Therefore, the number of accumulated electrons in the system is given by :

$$N(e)=\frac{\Delta A\boldsymbol{\cdot}V}{\epsilon\cdot l}$$

where *ΔA* and *V* stands for the absorbance change (0.57 at 600 nm) of the system by adding 50 μL 0.1 mmol/mL methyl viologen and the volume of the system (400 µL), respectively. *ϵ* of MV^+^$\boldsymbol{\cdot}$13500 L$\boldsymbol{\cdot}$mol^-1^$\boldsymbol{\cdot}$cm^-1^at 600 nm as referred in the main text, and $l$ is 0.2 cm.

$N\left( e \right)=\frac{\Delta A\boldsymbol{\cdot}V}{\epsilon\cdot l}$=$\frac{0.57\boldsymbol{\cdot}400\boldsymbol{\cdot}10^(-6)}{13500\cdot0.2}$=8.44 x 10^-8^ mol=8.44 x 10^-2^ µmol

The amount of MIP-177(Ti)-LT in the system is:

$m=\frac{3.2\boldsymbol{\cdot}400}{500}$=2.56 mg=2.56 x 10^-3^ g

The number of accumulated electrons per gram MIP-177(Ti)-LT is calculated as:

$n\left( e \right)=\frac{N(e)}{m}$=$\frac{8.44\boldsymbol{\cdot}10^(-2)}{2.56\boldsymbol{\cdot}10^(-3)}$ ≈ 33 µmol/g

**3.2. Photoaccumulated Electrons per Ti Atom in Dark Photocatalysis Experiment**

Since the H_2_ evolution yield is 300 µmol/g MIP-177(Ti)-LT, assuming 100% efficiency for electron transfer to the Pt co-catalyst and for the catalytic production of H_2_, the number of electrons stably accumulated in the system is 600 µmol/g MIP-177(Ti)-LT.

Given the molecular formula of MIP-177(Ti)-LT is reported as Ti_12_O_15_(mdip)_3_(formate)_6_ as referred in the main text, the molecular formula of MIP-177(Ti)-LT can be written as Ti_12_O_15_(C_17_H_12_O_8_)_3_(HCO_2_)_6_, and the molecular weight of MIP-177(Ti)-LT is 2117.01 g/mol.

Therefore, the moles of Ti atoms per gram MIP-177(Ti)-LT can be calculated as: $\frac{12}{2117.01}$=5.67 x 10^-3^ mol

The number of electrons in per Ti atom is: $\frac{600\boldsymbol{\cdot}10^(-6)}{5.67 \boldsymbol{\cdot}10^(-3)}$=0.10

The number of electrons in per Ti-oxo cluster is: 0.10 x 12= 1.2

**3.3. g Value Determination in EPR**

g value was calculated using the following equation:

g = (h·ν)/(μB·B) where:

h =Planck's constant =6.62 10^-34^ J·s

ν = frequency (Hz)

μB: Bohr magneton = 9.274 10^-24^ J/T

B: Magnetic field induction in Tesla

In our case, ν = 9.433 GHz = 9.433·10^9^ Hz

B_1_ (at resonance from Figure S7a) = 0.3355 T

B_2_ (at resonance from Figure S7a) = 0.3509 T

g_1_ = h·ν /μBΔB = (6.62 10^-34^·9.433·10^9^)/(9.274·10^-24^·0.3355) = 2.007

g_2_ = h·ν /μBΔB = (6.62 10^-34^·9.433·10^9^)/(9.274·10^-24^·0.3509) = 1.92

**3.4. Standardisation of Electrode Potentials to RHE in SEC**

In our system, 0.1 mol/L Na_2_SO_4_ was used as the electrolyte. Assuming a neutral aqueous environment where the pH=7, and neglecting the influence of hydrogen partial pressure, the electrode potential vs. Ag/AgCl reference can be converted to the estimated Reversible Hydrogen Electrode (RHE) using the following equation:

V_vs RHE_ = V_vs Ag/AgCl_ + V_Ag/AgCl vs SHE_ + 0.059 x pH

where V_Ag/AgCl vs SHE_ = +0.197 V (for saturated KCl), and pH=7, therefore, the estimated applied potential vs RHE can be calculated as:

V_vs RHE_ = V_vs Ag/AgCl_ + 0.610 V
